# Approximate Bayesian inference of directed acyclic graphs in biology with flexible priors on edge states

**DOI:** 10.1371/journal.pcbi.1014039

**Published:** 2026-03-16

**Authors:** Evan A. Martin, Venkata Patchigolla, Audrey Qiuyan Fu

**Affiliations:** 1 The Bioinformatics and Computational Biology Program, University of Idaho, Moscow, Idaho, United States of America; 2 The MD-PhD Program, Wayne State University School of Medicine, Detroit, Michigan, United States of America; 3 Department of Family Medicine and Public Health Sciences, Center for Molecular Medicine and Genetics, Institute for AI and Data Science, Wayne State University, Detroit, Michigan, United States of America; Fudan University, CHINA

## Abstract

Graphical models are widely used to represent dependence structures in biological systems, where directed edges may encode causal relationships under appropriate assumptions. We present baycn (BAYesian Causal Network), a novel approximate Bayesian method for inferring probabilities of edge directions and edge absence, while allowing flexible, user-specified priors to encode sparsity and an input graph to incorporate biological knowledge. For inference, we develop a Metropolis-Hastings-like sampler over graph structures based on a pseudo-posterior with a plug-in likelihood, which eliminates potentially high-dimensional nuisance parameters. This formulation substantially improves computational efficiency while yielding posterior probabilities that reflect Markov equivalence. We apply baycn to two genomic applications: distinguishing direct from indirect target genes of a shared genetic variant, and inferring combinatorial binding of transcription factors during tissue differentiation in Drosophila embryos. Both applications involve discrete and continuous data types that are common in genomics. Selected variables in these applications are treated as instrumental variables to help impose constraints on edge direction. Baycn demonstrates substantially improved accuracy at both the graph and edge levels, while existing methods do not handle mixed data, fail to capture weak signals, or are computationally infeasible.

## Author summary

Biological networks are widely used to describe relationships among genes, proteins, and other molecular features, but inferring which connections are real and which way information flows remains challenging. We present baycn, a Bayesian method that assigns probabilities to three possible states for each potential connection: A to B, B to A, or no edge. This edge-based representation allows researchers to incorporate prior biological knowledge, such as expected sparsity or known constraints. Baycn is designed to be computationally efficient, making it practical for real genomic studies. The estimated edge-state probabilities are well calibrated. It can analyze data that include both discrete and continuous values, which are common in genomics, and can use selected variables to improve inference of edge direction. Across simulations and two genomic applications, baycn achieves higher accuracy than existing approaches.

## Introduction

Graphical models (or networks), which may include directed and undirected edges, can be used to represent the statistical dependence among multiple variables and have wide applications in biology, such as gene regulatory networks [1,2] and protein-protein interaction networks [3,4]. Under appropriate assumptions, directed edges in a network may represent causal (or regulatory) relationships [5–7].

A key feature of graphs is their sparsity, which measures how many edges are present in a graph, or how likely an individual edge is expected to be present. We often have some knowledge about the sparsity of a graph, especially in biology where it remains a challenge to infer a reliable gene regulatory network due to complexity in biological processes and high dimension of biological variables involved [8,9]. For example, [10] estimated that there are about 6000 genes in the yeast genome, and that on average each gene is regulated by only 2.76 regulators (such as transcription factors), meaning that the probability of edge presence in the graph is around 2.76/6000 a priori. This example shows that we describe sparsity more easily at the edge level than for the whole graph, and that it is often of interest to infer the probability of a state of an edge: the two directions if present, and absence.

Here, we focus on Directed Acyclic Graphs (DAGs), also known as Bayesian networks, that have only directed edges and no directed cycles. Many Bayesian methods have been developed for DAG inference, which explore the graph space and draw a sample graph, either directly from a closed-form posterior distribution or more often use a Markov Chain Monte Carlo (MCMC) algorithm to sample the posterior distribution [11–34]. Most of these methods estimate the probabilities for edge directions and absence, although the priors are typically for all DAGs or for node orderings or partitions [18,19,24–26,28–30,32]. Such priors do not translate easily to priors on the states of an individual edge. For example, a uniform prior is often assumed for all the DAGs in these methods. In principle, one can calculate the overall probability of edge presence versus absence, dividing the total number of edges in all DAGs by the number of DAGs. The probability of each direction is then half the probability of edge presence. However, this calculation is not trivial, as the total number of DAGs is generally difficult to obtain. An exception is [33], which constructed an edge-level prior as a proxy for the prior on the entire graph. A few other Bayesian methods estimate the probability of the two directions only for the edges that are deemed present [13–15,17]. In particular, [15] used the prior above on gene regulators in their scanBMA method for gene expression data, but the probability of edge absence is not directly calculated. The posterior probabilities of the two possible directions are also treated as independent of each other, and can sum to above 1.

In our development, we formulate priors by considering three states for an edge: two directions and edge absence. We introduce a new Bayesian method that substantially eases the prior specification and estimates the posterior probabilities of three possible states of an edge. We represent a DAG by edge states (i.e., two directions and edge absence). This framework allows the user to specify a prior distribution for the edge states, which is easier to formulate with biological information and to specify the level of sparsity in the graph. We take a pseudo-Bayesian approach to designing the sampling algorithm for efficient inference, using a plug-in likelihood estimate for nuisance parameters. To reduce the size of the search space and speed up inference, our method can further take as input a graph from another more efficient graph inference method, e.g., constraint-based methods such as [35,36] and [37]. Although the same edge-state prior is applied across candidate edges, serving primarily as a sparsity-inducing regularization, posterior dependence among edges naturally arises through the likelihood and the additional structural constraints imposed by the input graph.

When instrumental variables are available, our method may be used for inferring more directed edges. In genomics, for example, if a genetic variant (denoted $T_{1}$, whose values are called genotypes) regulates the expression of a gene (denoted $T_{2}$), it can be used as an instrumental variable to better understand the regulatory relationships between $T_{2}$ and other genes, as the genotypes of individuals in a natural population are randomized; this idea underlies the Principle of Mendelian Randomization (PMR) [35,38,39]. Classical Mendelian randomization (MR) methods for causal effect estimation treat genetic variants as instrumental variables and rely on three standard assumptions [40–42]: (i) relevance: the genetic variant is associated with the gene expression of interest, (ii) independence: the variant is independent of unmeasured confounders due to random segregation of alleles, and (iii) exclusion restriction: the variant affects downstream genes only through the regulated gene, and not through alternative pathways. Consider the expression of another gene, denoted $T_{3}$. Under these assumptions, if $T_{3}$ is associated with changes in $T_{2}$, and no other processes can explain away this association, one may conclude that $T_{2}$ causally influences $T_{3}$, leading to the inference of a small graph $T_{1}\to T_{2}\to T_{3}$. However, our method does not require a genetic variant to satisfy the conditions of a genuine instrumental variable in the classical MR sense, nor does it aim to estimate causal effects. Instead, consistent with our previous work [35,38,39], we leverage the PMR only to impose the directional constraint that disallows edges pointing from gene expression back to genetic variants in the DAG. Thus, the PMR assumptions are used to reduce the space of admissible graph structures rather than as strict identification conditions.

We demonstrate the utility and performance of our method through extensive simulations and two representative genomic applications. The first application focuses on a central problem in statistical genetics: identifying which genes are directly regulated by a shared genetic variant versus indirectly affected through downstream regulatory cascades. Addressing this problem requires modeling complex dependency structures among molecular phenotypes (e.g., gene expression) while accounting for confounding effects due to other genes arising from genome-wide regulation. The second application examines combinatorial transcription factor (TF) binding during mesoderm development in Drosophila embryos, where multiple TFs bind DNA across developmental time points to jointly influence tissue differentiation. These binding profiles are highly correlated, making it difficult to disentangle direct regulatory relationships from indirect or redundant effects. Both applications involve mixed data types, including continuous molecular measurements and discrete genetic or tissue type information. Our method is designed to accommodate such mixed-type data by leveraging the PMR, and enables more accurate recovery of regulatory structure than existing approaches in these realistic settings.

## Methods

### A Bayesian graphical model for edge states

A DAG 𝒢=(𝐕,𝐄) is a set of vertices (nodes) 𝐕={1,2,...,b} and directed edges 𝐄⊆𝐕×𝐕, where 𝐕×𝐕 is the set of all ordered pairs of nodes, such as (j,k) that denotes an edge pointing from node *j* to node *k* where j,k∈𝐕. The (deterministic) adjacency matrix $A\in \{0,1{\}}^{b\times b}$ encodes the graph structure (or topology), where $A_{jk}=1$ and $A_{kj}=0$ represent the edge j→k, and $A_{jk}=0$ and $A_{kj}=1$ represent k→j. If $A_{jk}=A_{kj}=0$, there is no edge between nodes *j* and *k*.

We introduce baycn (BAYesian Causal Network), an alternate representation that describes the states of individual edges with the vector $S=(S_{1},S_{2},\ldots ,S_{m})$, where *m* is the number of edges. This vector may contain all the potential edges in the graph, or a subset based on prior knowledge. Note that the single subscript here denotes the index of the edge. The *i*th edge is between nodes *j* and *k* (without loss of generality, we will assume j<k) and is in state $S_{i}$, which can take on three values: $S_{i}=0$ if j→k, $S_{i}=1$ if k→j, and $S_{i}=2$ if the edge is absent. Let $p_{is}\equiv \mathrm{Pr}(S_{i}=s)$ for s∈{0,1,2}, so that $\sum_{s=0}^{2}p_{is}=1$. If $p_{i0}=p_{i1}$, we consider the edge bidirected with both directions being equally likely. When these edge-state probabilities are the same for all the edges, we drop the subscript *i* and refer to them as $p_{0}$, $p_{1}$ and $p_{2}$.

When we wish to emphasize the two nodes of an edge, we write $S_{jk}$ with the double subscript for nodes *j* and *k*. This notation connects the edge-state vector with the adjacency matrix:


$$\mathrm{Pr}(S_{jk}=0)=\mathrm{Pr}(A_{jk}=1),$$

$$\mathrm{Pr}(S_{jk}=1)=\mathrm{Pr}(A_{kj}=1),$$

$$\mathrm{Pr}(S_{jk}=2)=1-\mathrm{Pr}(A_{jk}=1)-\mathrm{Pr}(A_{kj}=1).$$

Given data observed at all *b* nodes, $T=(T_{1},T_{2},...,T_{b}{)}^{T}$, where each $T_{j}$ represents the random variable at node *j*, we aim to infer the posterior edge-state probabilities $\mathrm{Pr}(S_{i}=s|T)$ for all candidate edges *i* and states s∈{0,1,2} (these posteriors also sum to one for each *i*). It is convenient to summarize posterior information in a probabilistic adjacency matrix whose off-diagonal entries are $\mathrm{Pr}(A_{jk}=1|T)=\mathrm{Pr}(S_{jk}=0|T)$ and $\mathrm{Pr}(A_{kj}=1|T)=\mathrm{Pr}(S_{jk}=1|T)$; the posterior probability of absence is then the complement.

**Fig 1:**
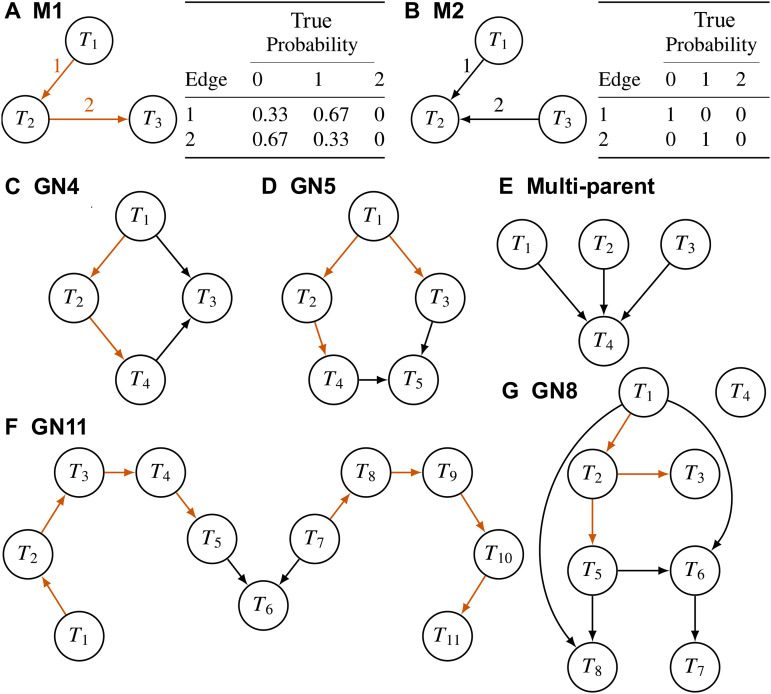
Seven topologies used in simulation studies. Orange edges have Markov equivalent edges and cannot be deterministically inferred. (**A**) A mediation model where $T_{2}$ is the mediator. (**B**) A v-structure. True probabilities on edge states account for Markov equivalence. The values show the existence of Markov equivalent graphs for M1 and no Markov equivalent graphs for M2. (**C**)-(**G**) Larger networks that contain M1 and M2 as subgraphs. See S1–S7 Figs for the true edge-state probabilities of the orange edges.

Edge-state probabilities need to account for Markov equivalence. Two DAGs are Markov equivalent if they have the same likelihood and represent the same conditional independence [43]. A set of Markov equivalent graphs form a Markov equivalence class. For example, the canonical mediation model of $T_{1}\to T_{2}\to T_{3}$ has two Markov equivalent graphs: $T_{1}\leftarrow T_{2}\to T_{3}$, and $T_{1}\leftarrow T_{2}\leftarrow T_{3}$ (Fig 1A). All three graphs depict marginal dependence between $T_{1}$ and $T_{3}$ (i.e., $T_{1}\text{⧸}\;⟂\;\;\;\;⟂T_{3}$) and conditional independence given $T_{2}$ (i.e., $T_{1}⟂\;\;\;\;⟂T_{3}\;|\;T_{2}$). Averaging over this equivalence class yields true edge-state probabilities (0.33,0.67,0) for the edge $T_{1}\to T_{2}$ and (0.67,0.33,0) for $T_{2}\to T_{3}$. In contrast, the v-structure $T_{1}\to T_{2}\leftarrow T_{3}$ (Fig 1B) has no Markov equivalent DAGs; it represents $T_{1}⟂\;\;\;\;⟂T_{3}$ and $T_{1}\text{⧸}\;⟂\;\;\;\;⟂T_{3}|T_{2}$ such that the true edge-state probabilities are 0 or 1.

The probability of the DAG with a known structure ***S*** and known parameters θ can be written as a product of conditional probabilities where each node is conditioned on its parents


$$\mathrm{Pr}(𝐓\;|\;S,\theta )=\prod_{j=1}^{b}\mathrm{Pr}(T_{j}\;|\;pa(T_{j}),\theta_{j}),$$ (1)

where $pa(T_{j})$ is the set of parent nodes of $T_{j}$, $\theta_{j}$ is the parameter vector for the distribution of $T_{j}$. When $pa(T_{j})=\emptyset$, where ∅ is an empty set, the probability reduces to $\mathrm{Pr}(T_{j}\;|\;\theta_{j})$.

When we assume normality for the continuous data (e.g., gene expression) at a node:


$$T_{j}\sim \text{N}(\mu_{j},\sigma_{j}^{2}),\;\;\text{and}\;\;\mu_{j}=\beta_{0j}+\sum_{k\in pa(T_{j})}\beta_{kj}T_{k},$$ (2)

where $\mu_{j}$ is the mean and $\sigma_{j}^{2}$ the variance. If there are no parents, $\mu_{j}=\beta_{0j}$.

Alternatively, for the binomial data (e.g., discrete genotype) at a node:


$$T_{j}\sim \text{Binomial}(n_{j},p_{j}),\;\;\text{and}\;\;\text{log}\left(\frac{p_{j}}{1-p_{j}}\right)=\beta_{0j}+\sum_{k\in pa(T_{j})}\beta_{kj}T_{k},$$ (3)

where $n_{j}$ is the number of trials (e.g., $n_{j}=2$ for genotype coded as 0/1/2, and $n_{j}=1$ for binary protein binding), and $p_{j}$ the “success” probability. Without parents, $\text{log}[p_{j}/(1-p_{j})]=\beta_{0j}$.

Together, these specifications define a conditional generalized linear modeling framework in which each node is modeled (with or without its parents) using a distribution appropriate to its data type.

### A Metropolis-Hastings-like sampler using pseudo-likelihood and the edge-state prior

We develop an iterative sampler that proposes changes to edge states and estimates the posterior probabilities, while accounting for Markov equivalence. Since our focus is on learning the graph structure rather than effect sizes of parent nodes on their child nodes, we view the parameters θ in Eq 1 as nuisance parameters.

For inference we adopt a pseudo-Bayesian approach based on classical results from likelihood theory. For each candidate graph ***S***, we use the maximum likelihood estimate (MLE) ${\hat{\theta }}_{S}$ for the nuisance parameter θ as the plug-in estimate to obtain the profile likelihood $L_{p}(S)=L(T|S,{\hat{\theta }}_{S})$. We then define a pseudo-posterior over graph structures,


$$\tilde{\pi }(S|T)\propto \pi (S)L_{p}(S),$$ (4)

where *π* is the prior, and $\tilde{\pi }$ is the pseudo-posterior that is derived from replacing the full marginal likelihood ∫L(T|S,θ)π(θ|S)dθ with a pseudo-likelihood. This approach follows the general framework where a pseudo-likelihood is combined with a prior to enable inference when nuisance parameters are high-dimensional or direct integration is computationally prohibitive; in our implementation, the pseudo-likelihood is taken to be the profile likelihood (see the reviews in [44] and [45]).

Theoretical justification for this approach follows from properties of the profile likelihood. Although profile likelihood is not a genuine likelihood, its theoretical properties are well understood. Under standard regularity conditions, profile likelihood is second-order asymptotically Bayesian with respect to an implicit conditional prior on the nuisance parameters given the parameter of interest (see Proposition 4.1 in [46]). Being second-order asymptotically Bayesian means that the pseudo-likelihood and a genuine integrated likelihood have the same local behavior around the true parameter of interest. Here, the integrated likelihood refers to the likelihood obtained by integrating out the nuisance parameters with respect to the conditional prior. Local behavior is defined with respect to an $n^{-1/2}$-local neighborhood of the true parameter, corresponding to the rate at which likelihood-based estimators such as the MLE fluctuate asymptotically. In this neighborhood, the change in the pseudo-likelihood agrees with that of the integrated likelihood up to terms of order $O_{p}(n^{-1})$, which means that the discrepancy between the two log-likelihoods vanishes at rate $n^{-1}$ in probability.

As a consequence, inference based on the profile likelihood yields the same first- and second-order asymptotic behavior as inference based on the integrated likelihood, including consistency and asymptotic normality. Any bias introduced by profile likelihood diminishes asymptotically. These results provide theoretical support for the use of pseudo-likelihood–based inference in large-sample settings. Here, we show in simulations that this yields accurate estimates of edge-state probabilities while avoiding the computational burden of sampling θ.

Following [15,33], we use a prior that assumes all the edges to be independent: $\mathrm{Pr}(S)=\prod_{m}\mathrm{Pr}(S_{i}=s_{i})$. We can construct the prior based on our knowledge or belief about graph sparsity. For example, as noted in the Introduction, in the yeast genome we expect the prior probability for each direction to be 2.76/6000/2, and for edge absence 1-2.76/6000. Although the construction of Pr(S) treats the edges independently, the pseudo-posterior induces dependence among the edges through the likelihood.

The input of our algorithm is the binary adjacency matrix of a candidate graph and the data at the nodes. The candidate graph may be a fully connected graph, where all nodes are connected. A more efficient approach is to run a fast graph inference algorithm to produce a candidate graph and use it as the input, even if this graph contains false edges.

At the *t*th iteration, the key steps of the Metropolis-Hastings-like algorithm are as follows (also see Algorithm 1):

1. Generate a proposal graph $S_{t+1}^{\prime }$ from the current graph $S_{t}$, removing directed cycles in the proposal. When t=1, the current graph $S_{1}$ is initialized by randomly selecting a graph from the graph space defined by the input graph.
2. Calculate the acceptance probability $\alpha_{t}$:
  $$\alpha_{t}=\min\{\frac{\mathrm{Pr}({𝐒}_{t+1}^{\prime })\mathrm{Pr}(𝐓\;|\;{𝐒}_{t+1}^{\prime },\theta_{t+1})\mathrm{Pr}({𝐒}_{t}\;|\;{𝐒}_{t+1}^{\prime })}{\mathrm{Pr}({𝐒}_{t})\mathrm{Pr}(𝐓\;|\;{𝐒}_{t},\theta_{t})\mathrm{Pr}({𝐒}_{t+1}^{\prime }\;|\;{𝐒}_{t})},\;1\},$$ (5)
  where Pr(S) is the prior probability of the edge states, Pr(T|S,θ) the graph pseudo-likelihood, and $\mathrm{Pr}(S\;|\;S^{\prime })$ or $\mathrm{Pr}(S^{\prime }\;|\;S)$ the transition probability. Here, we estimate θ by maximum likelihood estimation given the corresponding graph.
3. Generate a random probability *u* from the uniform distribution U(0,1). Accept the proposal and set ${𝐒}_{t+1}={𝐒}_{t+1}^{\prime }$ if $u<\alpha_{t}$; or stay at the current graph and set ${𝐒}_{t+1}={𝐒}_{t}$ otherwise.

**Algorithm 1. Metropolis–Hastings-like sampler with cycle resolution.**

1: Initialize $S_{1}$ as a DAG drawn from the input candidate graph space

2: **for** t=1,…,T−1 **do**

3:  Propose a candidate graph $S_{t+1}^{\prime }$ from the current graph $S_{t}$

**while** $S_{t+1}^{\prime }$ contains directed cycles **do**

Identify a strongly connected component (SCC) with ≥2 nodes

Modify an edge within the SCC

**end while**

4:  Compute acceptance probability $\alpha_{t}$

5:  **if** $Uniform(0,1)<\alpha_{t}$ **then**

6:   $S_{t+1}\leftarrow S_{t+1}^{\prime }$

7:  **else**

8:   $S_{t+1}\leftarrow S_{t}$

9:  **end if**

10: **end for**

To generate the proposal graph in Step 1, we first determine the number of edges to change states by sampling from a binomial distribution Bin(m,1/m), where 1/m is the “success” probability. For each of the selected edges, we then sample from a Bernoulli distribution with probability *p* to decide which edge state to change to. Since we do not allow for an edge to remain in the same state, *p* is determined by the prior of the two remaining edge states. For example, if an edge in state 0 is selected to change states, and if the prior probability for the edge states is $\left(p_{0},p_{1},p_{2})=(0.05,0.05,0.9\right)$, then the probability of switching to state 1 is p=0.05/(0.05+0.9).

To detect and remove directed cycles in the proposal graph, we exploit the characterization of cycles via strongly connected components (SCCs). In a directed graph, an SCC is defined as a maximal set of nodes such that for every pair of nodes *j* and *k* in the set, there exists a directed path from *j* to *k* and a directed path from *k* to *j* [47]. Results in [47] imply that a directed graph is acyclic if and only if all of its SCCs consist of a single node, and that every directed cycle is fully contained within a single SCC. Accordingly, at each proposal we decompose the graph into SCCs using an existing linear-time implementation (the components function with mode = “strong” in the R package igraph), identify components containing two or more nodes, and randomly modify an edge whose endpoints lie within such a component. This procedure is repeated until all SCCs are singletons, yielding an acyclic directed graph that serves as the candidate graph.

The binomial and Bernoulli probabilities in Step 1 are then used to calculate the transition probability in Step 2. We show that the transition probabilities do not depend on the path taken from the current graph to the proposed graph (the process of introducing and removing directed cycles) but only on the edges that have different states between the two graphs:

**Theorem 1.** *When calculating the acceptance probability, the transition probabilities between the current graph* ***S*** *and proposed graph* $S^{\prime }$, $\mathrm{Pr}(S^{\prime }|S)$ and $\mathrm{Pr}(S|S^{\prime })$, *depend only on the edges whose states are different between the two graphs.*

*Proof.* See the proof in Note A in S1 Text and examples in Note B in S1 Text.

**Fig 2:**
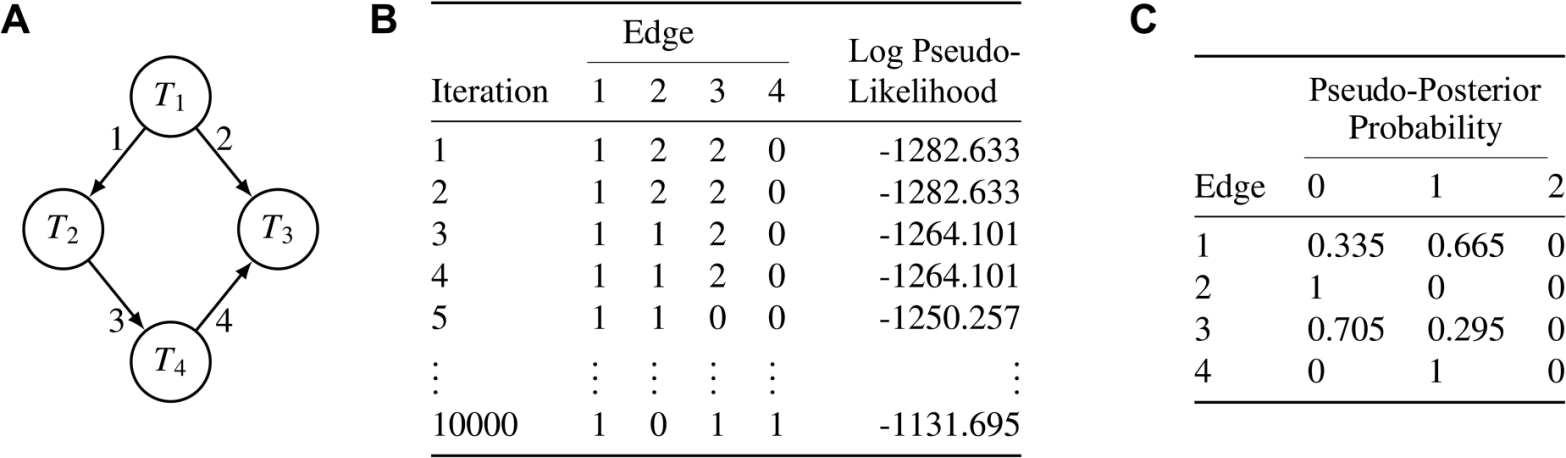
An example of the output from baycn. (**A**) The true graph GN4. The candidate graph used for inference will also consider only these four edges. (**B**) Edge states and log pseudo-likelihood for the graph accepted at each iteration of the Metropolis-Hastings-like algorithm. (**C**) The proportion of each edge state in the sample provides an estimate of the pseudo-posterior probability of the edge state.

This algorithm generates a sample of graphs represented by edge states (Fig 2). For each edge, the relative frequencies of the three states in the sample provide an estimate of the pseudo-posterior $\tilde{\pi }(S|T)$.

Through changes in edge states, our algorithm can sample from multiple Markov equivalent graphs and thus produce posterior probabilities that account for Markov equivalence. With sufficient data, the posterior probabilities of edge states should be the same asymptotically as expected under Markov equivalence.

Under the PMR, instrumental variables are restricted from having incoming edges from other nodes. We enforce this directional constraint in the proposal step of our graph sampler. For example, consider an undirected network $T_{1}-T_{2}-T_{3}$, which admits four possible directed networks: (i) $T_{1}\to T_{2}\to T_{3}$, (ii) $T_{1}\leftarrow T_{2}\leftarrow T_{3}$, (iii) $T_{1}\leftarrow T_{2}\to T_{3}$, and (iv) $T_{1}\to T_{2}\leftarrow T_{3}$. If $T_{1}$ is designated as a potential instrumental variable, then any existing edge between $T_{1}$ and $T_{2}$ is constrained to point from $T_{1}$ to $T_{2}$, and only graphs (i) and (iv) remain admissible. These two graphs have different likelihoods and are therefore distinguishable. In this simple example, our algorithm can propose only two moves for the edge $T_{1}-T_{2}$ ($T_{1}\to T_{2}$ or edge absence) while allowing three moves for the unconstrained edge $T_{2}-T_{3}$ ($T_{2}\to T_{3}$, $T_{2}\leftarrow T_{3}$, or edge absence). The implementation is equivalent to imposing a blacklist of edges by other methods.

### Relationship to existing Bayesian methods

We compare baycn to six methods (Table 1). Five of them are Markov chain Monte Carlo (MCMC) methods, and scanBMA [15] is not based on sampling. Two MCMC methods are based on the graph structure: Gibbs [24] and MC^3^ [18], and two methods are based on node orderings or node partitions (i.e., subsets of nodes): order MCMC [19,32] and partition MCMC [26,32]. These methods assign a prior to the graph structures or node orderings/partitions of the entire graph. Similar to our method, BCDAG [31,34] also assigns a prior to each possible edge, although it infers not only the graph structure, but also the parameters, with a focus on Gaussian data.

**Table 1:** Summary of our method and other Bayesian methods for network inference.

	baycn	Gibbs [24]	MC^3^ [18]	order MCMC [19,32]	partition MCMC [26,32]	BCDAG [31,34]	scanBMA [15]
Input	Data matrix, adjacency matrix, prior on edge states	Data matrix, edge list (optional), prior on graph space	Data matrix, edge list (optional), prior on graph space	Data matrix, edge list (optional), parameters for graph score	Data matrix, edge list (optional), parameters for graph score	Data matrix, prior for edge presence	Data matrix, prior on each edge
Output	Posterior probabilities of three edge states	Posterior probabilistic adjacency matrix	Posterior probabilistic adjacency matrix	Posterior probabilistic adjacency matrix	Posterior probabilistic adjacency matrix	Posterior probabilistic adjacency matrix	Posterior probabilities of parent nodes
Sampling Space	Candidate edges and their direction	Parent nodes	Candidate edges and their direction	Node orderings	Node partitions	Candidate DAGs and parameters	Not applicable
Sampling Steps	Change states for ≥1 edges	Change parents for ≥1 nodes	Add or remove an edge	Move a node to a new position	Split or merge existing partitions	Closed form for posterior	Not applicable
Type of Prior	Edge states	Parameters and graph structure	Parameters and graph structure	Parameters and node orderings	Parameters and node partitions	Parameters and graph structure	Parameters and edges
Type of Data	Discrete, continuous, or mixed	Discrete or continuous	Discrete or continuous	Discrete or continuous	Discrete or continuous	Continuous	Continuous
R Package	baycn	structmcmc	structmcmc	BiDAG	BiDAG	BCDAG	networkBMA

ScanBMA [15] is a Bayesian model averaging approach that assigns prior probabilities to individual regulator-target pairs [13–15] and performs deterministic model search for each node independently. As a result, scanBMA estimates posterior probabilities for the two possible directions of an edge independently, so the two probabilities may sum to more than one. It also does not provide an explicit posterior probability for edge absence.

### Simulation study

We performed the simulation study to assess correctness of the software implementation, to study the choice of priors, and compare baycn to other methods in controlled settings. We simulated data under seven different topologies (Fig 1). Each node was simulated under a linear model in Eq 2 with the variance set to 1 and the intercept $\beta_{0}$ set to 0. All the other coefficients take the same value, which is referred to as the signal strength. We considered three levels of the signal strength: 0.2 (weak), 0.5 (moderate), and 1 (strong), and three levels of the sample size: 100, 200 and 600. For each topology, we simulated 25 datasets under each of the nine combinations of signal strength and sample size. When summarizing the results, if the results are grouped by, for example, topology, we use the output from all the datasets with different sample sizes and signal strengths.

We evaluate the performance using the following metrics:

1. The edgewise Mean Squared Error (eMSE): This is the MSE between the true probabilities and posterior probabilities of the three states for edge *i*:
  $${\text{eMSE}}_{i}=\frac{1}{3}\sum_{s=0}^{2}[\mathrm{Pr}(S_{i}=s{)}^{\text{true}}-\mathrm{Pr}(S_{i}=s\;|\;𝐓){]}^{2},$$ (6)
  where $\mathrm{Pr}(S_{i}=s{)}^{\text{true}}$ is the true edge-state probability under Markov equivalence. This metric informs us which edges are more accurately inferred and which ones are not. Note that the true probabilities can be calculated only when the structure of the true graph is known: we identify all possible graphs in the Markov equivalence class of the true graph, and then calculate the frequency of an edge being in each of the three states (see S1–S7 Figs).
2. MSE_1_: This is the MSE for the whole graph based on three possible edge states. It is the eMSE averaged over all *m* edges in the graph:
  $${\text{MSE}}_{1}=\frac{1}{m}\sum_{i=1}^{m}{\text{eMSE}}_{i}=\frac{1}{3m}\sum_{i=1}^{m}\sum_{s=0}^{2}[\mathrm{Pr}(S_{i}=s{)}^{\text{true}}-\mathrm{Pr}(S_{i}=s\;|\;𝐓){]}^{2}.$$ (7)
3. MSE_2_: This is the MSE between the true and posterior probabilistic adjacency matrices on all *m* edges. In other words, this metric uses the probability only of the two edge directions.
  $$\begin{array}{c} \begin{array}{cc} {\text{MSE}}_{2}=\frac{1}{2m}\sum_{\begin{array}{c} (j,k)\;\text{or}\\ \;(k,j)\in 𝐄 \end{array}} & \;\{[\mathrm{Pr}(A_{jk}=1{)}^{\text{true}}-\mathrm{Pr}(A_{jk}=1\;|\;𝐓){]}^{2}\\ & \;\;+[\mathrm{Pr}(A_{kj}=1{)}^{\text{true}}-\mathrm{Pr}(A_{kj}=1\;|\;𝐓){]}^{2}\}. \end{array} \end{array}$$ (8)
4. Precision and power for the whole graph. Precision, or 1−False Discovery Rate (FDR), measures how many of the inferred edges are true, and power (or recall) measures how many of the true edges are correctly inferred. For calculation, we apply a cutoff of 0.5 to the posterior probability of edge presence. These metrics ignore the nuances in the probabilities, but are easy to interpret and provide a quick indication of the inference accuracy.

## Results

### Results from simulation studies

#### Posterior probabilities of edge states are calibrated.

We ran baycn once per simulated dataset, used a burn-in of 20%, and used a sparse prior, $\left(p_{0},p_{1},p_{2})=(0.05,0.05,0.9\right)$, on edge states. For M1, M2, GN4, GN5, and multi-parent topologies (Fig 1A–1E), we ran baycn for 30,000 iterations. For GN8 and GN11 (Fig 1F–1G), we ran baycn for 50,000 iterations. We retained 200 samples after burn-in in all the runs. Trace plots of the sample graphs and their log pseudo-likelihoods did not show signs of poor mixing (see S8 Fig for selected trace plots for GN4, GN8, GN11 across sample sizes and signal strengths).

As expected when using the true graph as the input, MSE_1_ (based on three edge states) decreases as *β* and *N* increase for each topology, and is particularly affected by *β* (S1 Table). When MSE_1_ < 0.1, the inference is typically accurate; it means that the direction of each edge is correctly inferred, and the posterior probability of each edge state is similar to the true one. Using this cutoff, we observed that baycn performs well in general when the signal strength is not weak. For both M2 and multi-parent topologies that only contain v-structures, however, MSE_1_ is much larger at β=0.2. This is consistent with previous observation that it is generally difficult for existing graph inference algorithms to correctly identify v-structures with a weak signal [35,38].

#### Sparse prior lowers false positives while retaining power.

For this assessment we included a false edge in M1, M2 and GN4, and two false edges in GN11 (S9 Fig). We used the same data generated in the previous section for these topologies (without false edges) and ran baycn with the input being the true edges plus the false edges. The user can specify their prior belief on sparsity through the prior edge-state probabilities. Here, we explored the impact of three edge-state priors on the inference, with an increasing probability of edge absence: prior 1: $\left(p_{0},p_{1},p_{2})=(1/3,1/3,1/3\right)$; prior 2: $\left(p_{0},p_{1},p_{2})=(0.25,0.25,0.5\right)$, and prior 3 (sparse prior): $\left(p_{0},p_{1},p_{2})=(0.05,0.05,0.9\right)$.

We calculated the eMSE for each edge and again used <0.1 as the cutoff. In all four graphs, increasing $p_{2}$ (thus making the graph more sparse) consistently reduced eMSE for the false edges, while preserving accurate estimates of edge probabilities for the true edges (S2–S5 Tables). In particular, prior 3 reduces the eMSE on false edges by an order of magnitude, compared to the other two priors, demonstrating its ability to balance the need to detect false positive edges and to correctly infer true edges. We therefore used the sparse prior in subsequent method comparisons and in applications.

#### Competitive performance when compared with other Bayesian methods.

To compare baycn with other methods, we focus on GN4, GN11 and GN8, which have different levels of complexity (Fig 1). Using a fully-connected network as the input to all the methods, we ran each sampling-based method for 30,000 iterations on GN4, and for 50,000 iterations on GN8 and GN11. We used a burn-in of 20% and retained 200 samples in all the runs.

We first benchmarked runtimes of these methods on an Intel Xeon D-1540 (2.00 GHz processor, 128 GB of RAM) using GN4, GN8, and GN11 with β=1 and N=600 (Table 2). Across all three topologies, scanBMA was the fastest, and Gibbs the slowest. baycn had a comparable runtime to partition MCMC. Since Gibbs was substantially slower than other methods, we excluded it from further comparisons.

**Table 2:** The mean runtime in seconds across 25 datasets. For each topology, 25 datasets were generated with β=1  and N=600. Each algorithm was run once per dataset, and the runtime in seconds was recorded. All methods were run on an Intel Xeon D-1540 (2.00 GHz processor, 128 GB of RAM).

	Runtime (seconds)						
Topology	baycn	Gibbs	MC^3^	order	partition	BCDAG	scanBMA
GN4	6.50	235.00	11.94	1.78	3.93	38.24	0.02
GN8	11.47	363.91	22.49	2.88	7.97	150.65	0.05
GN11	12.65	380.97	23.52	3.10	9.39	237.63	0.09

We calculated precision, power and MSE_2_ (between the true and posterior probabilistic adjacency matrices) to assess the inference accuracy. Note that MSE_2_ does not apply to scanBMA, as the posterior probabilities from scanBMA have a different interpretation from the other methods (see section “Relationship to existing Bayesian methods”).

**Fig 3:**
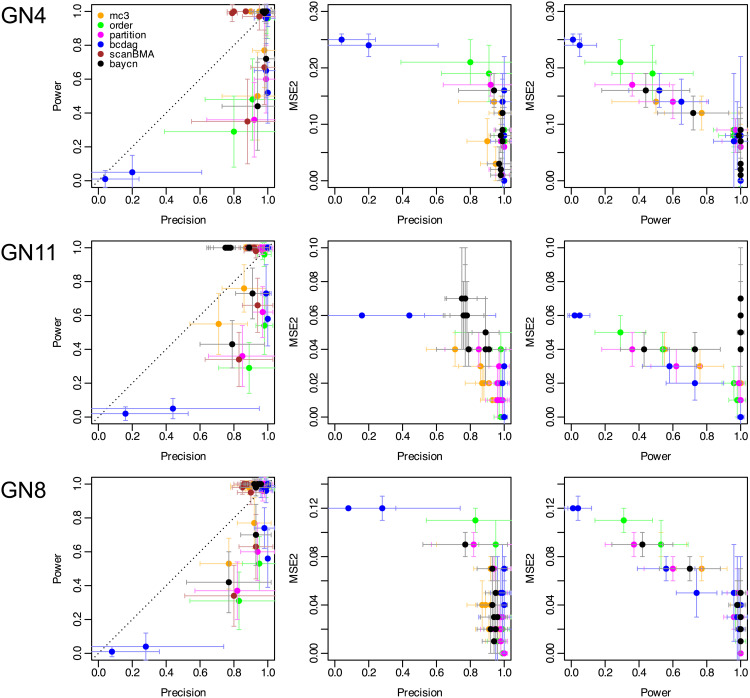
Precision, power, and MSE_2_ (on the posterior probabilistic adjacency matrix) for baycn and other Bayesian methods on simulated data with varying sample sizes and signal strengths from GN4, GN11 and GN8. A fully connected graph was used as the input to each method. For each method, we considered nine scenarios (all combinations of three sample sizes and three signal strengths) and simulated 25 independent datasets in each scenario. After applying the methods, we calculated the mean and standard deviation of each metric in each scenario. Therefore, in each plot here, every method has nine dots, all represented by one color. Each dot is the mean of a metric, and the whiskers on either side of the dot are one standard deviation of that metric. MSE_2_ was not calculated for scanBMA, since the posterior probabilities from scanBMA have a different interpretation from the MCMC methods (see section “Relationship to existing Bayesian methods”).

Across these Gaussian datasets, most methods perform similarly in most scenarios, with MC^3^ and baycn generally leading (Fig 3; S6 Table). Performance of each method improves with larger sample size or stronger signal strength. When the data is less informative (smaller sample size or lower signal strength), BCDAG degrades markedly (e.g., on GN4, mean precision = 0.04; mean power = 0.01; S6 Table). Order and partition MCMC also show reduced mean precision in these settings (on GN4 and GN11: 0.29 for order MCMC and 0.36 for partition; on GN8: 0.31 for order MCMC and 0.37 for partition MCMC; S6 Table).

Taken together with the runtime results, baycn combines a coherent modeling framework and inference accuracy with competitive runtime, offering a favorable trade-off between speed and accuracy.

### Applications to genomic data

Simulations on Gaussian data verified the correctness of baycn and showed competitive performance across multiple graph topologies. Real genomic datasets, however, are noisier and typically combine mixed data types (e.g., continuous phenotypes and discrete genotypes). In this section, we applied baycn and other methods to these real datasets. An overview of the analysis workflow using baycn, including data preprocessing and confounding variable identification, is shown in (S10 Fig). Gibbs was omitted due to its high runtime, and MC^3^ was also omitted because the available R implementation failed on mixed-type inputs. As summarized in Table 1, apart from baycn, none of the other implementations natively handle mixed data; when used in the two applications here they treat all variables as continuous.

#### Case study A: Causal network inference of transcription regulation with genetic variants.

Genetic variants play an important role in regulating gene expression [48]. The GEUVADIS (Genetic European Variation in Disease) project identified a large number of variants that are associated with the expression of one or more genes in Lymphoblastoid Cell Lines (LCLs) in Europeans and Africans [49]. Among these variants, which are termed expression quantitative trait loci (eQTLs), 62 are associated with more than one gene. However, since the association analysis examined one eQTL-gene pair at a time, it is unclear which associated genes are more likely to be direct targets (i.e., having an edge with the variant), and which ones indirect targets (i.e., not having an edge with the variant).

To address this question, we infer graphs for these eQTL-gene sets, treating each eQTL as an instrumental variable under the PMR [50]. Under this principle, an edge connecting an eQTL and a gene points only to the gene, but not the other way around, since the DNA (eQTL here) regulates the RNA (expression). We focus on the larger European sample of 373 individuals. The gene expression data in GEUVADIS had been normalized using the PEER method [51] to remove potential impact of demographic variables, batch effect, and other covariates. To account for confounding from genes outside the small graph, we performed principal component analysis on the gene expression from all genes in the GEUVADIS data. We used Holm’s method to control the familywise type I error rate across all the p-values at 5% [52], and identified principal components (PCs) that are highly significantly associated with the eQTLs or genes in all the trios of that tissue. These PCs were then included in the network as confounding variables. The eQTL-gene sets Q8, Q21, Q23, Q37, and Q50 all have at least one PC associated with them while the eQTL-gene sets Q20 and Q62 do not have any PCs associated with them.

On each eQTL-gene-PC set, we used a fully connected graph as the input to baycn with a sparse prior $\left(p_{0},p_{1},p_{2})=(0.05,0.05,0.9\right)$. The input graph did not contain edges among PCs, as they are independent by definition. In this application, baycn models genotype nodes (which are instrumental variables) as binomial variables, and gene expression nodes and PCs as Gaussian variables. Since there is no ground truth, we performed three long runs each of 500,000 iterations from a different random starting point with 20% burn-in and 1,000 retained samples. Trace plots of the sampled graphs and their log pseudo-likelihoods did not show signs of poor mixing (S11 Fig), and the three runs produced qualitatively identical results. In fact, results from multiple runs of 50,000 iterations were already able to produce the same results. We averaged the posterior probabilities from three long runs for final inference.

**Fig 4:**
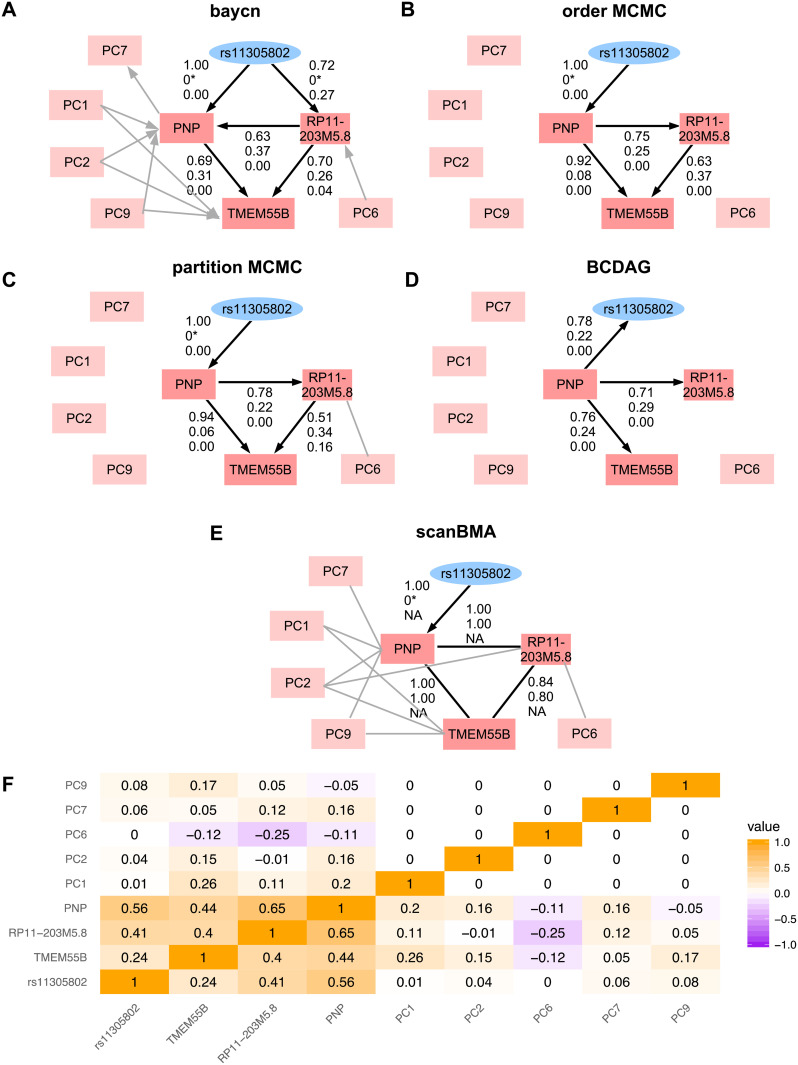
Inference of the GEUVADIS eQTL-gene set Q8. This eQTL is associated with three genes. (**A**)–(**E**) Graphs inferred by different methods with posterior probabilities shown only for edges of biological interest. The three probabilities are for the displayed direction, the opposite direction, and edge absence, respectively. 0* indicates that the corresponding direction was blacklisted during inference. BCDAG in (**D**) does not allow edge blacklist. scanBMA in (**E**) is unable to infer the probability of edge absence (hence the NAs) or distinguish the two directions. (**F**) Heatmap of Pearson correlations in the data. The posterior probability of all the edges from different methods are in S7–S11 Tables.

We further ran order MCMC, partition MCMC, BCDAG, and scanBMA on the eQTL-gene set Q8 from GEUVADIS to compare with baycn (Fig 4). We used the same fully connected graph as input for order and partition MCMC, and applied the same constraint that a gene cannot be the parent of an eQTL. Although BCDAG and scanBMA did not take an input graph or allow edge exclusion, they allow for a prior probability on edge presence, which we set to be 0.1, same as our sparse prior for baycn. As with baycn, we ran each sampling-based method for 500,000 iterations with 20% burn-in and 1,000 retained samples. We set the posterior probability cutoffs for edge presence to be 0.5, and considered an edge directed if the difference between the posterior probabilities for the two directions is greater than 0.2.

Inferred graphs with the posterior probabilities are shown in Fig 4 (see tables of posterior probabilities for all edges from different methods in S7–S11 Tables). The inference from baycn, order MCMC and partition MCMC is similar for most of the edges among the eQTL and the genes, except that baycn is the only method that identifies an edge between the eQTL and RP11-203M5.8, which is consistent with the relatively strong correlation between the two (Fig 4A–4C). BCDAG also missed an edge between two genes. Moreover, the other three methods missed nearly all the edges with the PCs, suggesting their insensitivity to weaker correlations (Fig 4E). On the other hand, scanBMA is as sensitive to correlations as baycn is, but cannot infer edge absence or direction (Fig 4D).

The inference of the direction of the edge between PNP and RP11-203M5.8 is different under baycn and the other three sampling-based methods (Fig 4A–4D). This difference is likely driven by whether the PCs are accounted for. Specifically, when regressing RP11-203M5.8 on all the other variables, PC2 has a significantly nonzero coefficient (*p*-value: 0.001), which means that RP11-203M5.8 and PC2 are conditionally dependent given PNP and other variables. On the other hand, RP11-203M5.8 and PC2 are marginally independent (correlation: -0.01) of each other, but both have moderate to strong correlation to PNP (Fig 4E), suggesting that the conditional dependence between RP11-203M5.8 and PC2 is induced by PNP, thus forming a v-structure: RP11-203M5.8 → PNP ← PC2. This is why baycn infers an edge from RP11-203M5.8 to PNP. To further verify the impact of PCs on inference, we applied baycn to Q8 but excluded the PCs. This analysis led to the same inference as order/partition MCMC, which effectively ignored the PCs (S12 Table). These results demonstrate the importance of accounting for confounding variables and that of having sufficient sensitivity to the signals in data. Analysis of the other eQTL-gene-PC sets by baycn also showed consistency between posterior probabilities and correlations (S12–S17 Figs; S13–S18 Tables).

#### Case study B: Inferring combinatorial binding of transcription factors in tissue differentiation.

**Fig 5:**
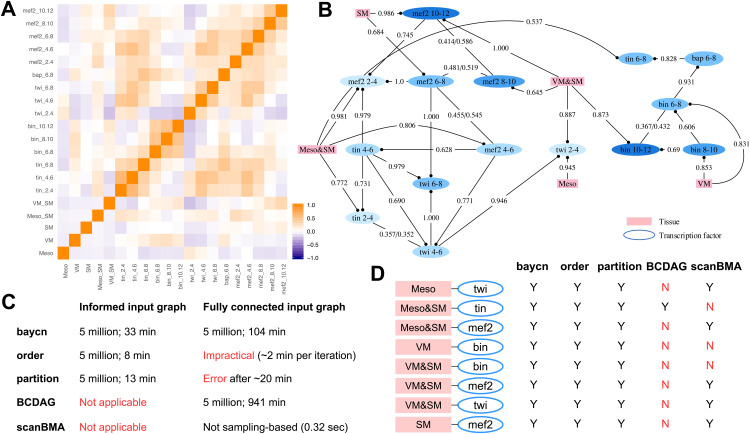
Combinatorial binding of transcription factors (TFs) in five tissue types from the benchmark dataset on Drosophila embryo. [54]. The TFs are: Twist (Twi), Tinman (Tin), Myocyte enhancing factor 2 (Mef2), Bagpipe (Bap), and Biniou (Bin). The tissue types are: mesoderm (Meso), mesoderm and somatic muscle (Meso&SM), visceral muscle (VM), visceral muscle and somatic muscle (VM&SM), and somatic muscle (SM). (**A**) The heatmap of Pearson correlations following hierarchical clustering. (**B**) The graph inferred by baycn. To avoid confusion when interpreting directed edges in time-series data, we use a dot in place of an arrow. Except for bidirected edges, only the inferred direction with the corresponding posterior probability is shown. Posterior probabilities were averaged over three independent runs of 5 million iterations. Shades of the TFs reflect the timing of the TF binding: later time points correspond to darker shades. (**C**) Computational feasibility of each method with different input graphs. Total runtime (in minutes; on CPUs of a shared computing cluster) is reported for the four sampling-based methods over 5 million iterations. Runtime for baycn is the average from three independent runs. (**D**) Known relationships between tissues and TFs with the corresponding presence/absence inference from each method. A relationship is considered to be present if a method infers at least one edge between any of the time points for a given TF and its associated tissue.

Transcription factors (TFs) regulate the expression of target genes by combinatorial binding to regulatory sequences in the genome [53]. Here, we re-analyzed a set of highly correlated TF binding profiles measured in 310 *cis*-regulatory modules (CRMs, which are DNA sequences) during early development in Drosophila [54] (Fig 5A). These TFs play central roles in Drosophila mesoderm differentiation, and have numerous regulatory interactions that are well supported by experimental evidence [54,55]. The dataset, previously analyzed using a non-Bayesian graphical model approach for each of five tissues [55], consists of mixed data: binary indicators of whether a CRM is expressed in one of five tissue types, and continuous ChIP-chip binding measurements of five key TFs in these CRMs at two-hour intervals during mesoderm development in these tissue types in the embryos of *Drosophila melanogaster*. The five tissue types are mesoderm (Meso), somatic muscle (SM), visceral muscle (VM), mesoderm and somatic muscle (Meso&SM), visceral muscle and somatic muscle (VM&SM). The five TFs include Twist (Twi), Tinman (Tin), Myocyte enhancing factor 2 (Mef2), Bagpipe (Bap), and Biniou (Bin). Because of repeated measurements over time and combinatorial binding, there exist strong correlations among these binding profiles, and most graph inference methods could not tell them apart and tended to infer a dense network [55].

We treated the binary tissue types as instrumental variables to restrict edges involving tissue types to a single direction. Since tissue types function as labels and not mechanistic variables, edge direction here represents direct, unmediated association rather than underlying biological processes. We used a machine learning network inference method MRPC [35,38] to generate an initial input graph from these mixed data, and included additional edges to reduce the risk of excluding true relationships (S18 Fig). Here, baycn models binary tissue types (which are instrumental variables) as binomial variables, and continuous TF binding profiles as Gaussian variables. We performed three long runs each of 5 million iterations from a different random starting point. The burn-in remained 20% and we retained 1,000 samples after burn-in. Trace plots of the sampled graphs and their log pseudo-likelihoods did not show signs of poor mixing, either (S19 Fig). We averaged the posterior probabilities from three long runs for final inference. Also similar to before, an edge is considered directed if the difference between the posterior probabilities for the two directions is greater than 0.2.

Our analysis showed that baycn can identify TFs known to drive tissue differentiation (Fig 5B; S19 Table). The inferred edges are consistent with the known relationships (Fig 5C). However, it is worth emphasizing that edges in a DAG represent conditional dependence rather than temporal or regulatory direction. For example, the inferred edge [Twi at 4-6h → Twi at 2-4h] does not imply that later binding regulates earlier binding. Instead, the configuration [Twi at 4-6h → Twi at 2-4h ← Meso&SM] indicates that the effect of Twi binding at 4-6h is not marginally related to Meso&SM, but is conditionally dependent of this tissue type given Twi binding at 2-4h. Similarly, in the subgraph for SM, we have [SM → Mef2 at 10-12h → Mef2 at 2-4h] and [SM → Mef2 at 6-8h → Mef2 at 2-4h]. This subgraph indicates that the association between Mef2 binding at earlier hours and SM can be explained away by the binding of Mef2 after 6 hours, meaning that the formation of SM is more directly associated with later Mef2 binding than with earlier binding.

The inferred network recapitulates several well-established regulatory relationships in mesoderm and muscle development. Specifically, we confirmed the edge connecting a Twi node with Meso, consistent with the established role of Twi as a primary TF required for mesoderm formation [56]. Twi is also known to directly regulate the expression of both Tin and Mef2 [57], and the corresponding inferred edges align with these experimentally validated regulatory relationships. Additionally, Tin and Mef2 play essential roles in dorsal mesoderm specification [58,59] and muscle tissue differentiation [60–62]; concordantly, our inferred network contains edges linking Meso&SM with both TFs. Furthermore, Bap and Bin, which are expressed only in visceral muscle [63,64], form a distinct subgraph in our inferred graph and are connected only to VM or VM&SM. Notably, even when a fully connected graph was provided as input (with tissue types treated as instrumental variables; three independent runs each with 5 million iterations, 20% burn-in and 1,000 retained samples), baycn did not infer any edges between these TFs and non-VM tissue types (S20 Fig; the posterior probabilities of edge absence are high, ranging from 0.756 to 0.900), despite the two TFs not being grouped together in the correlation heatmap (Fig 5A). These results suggest that the inferred network structure reflects biologically meaningful specificity rather than artifacts of the input graph.

We further evaluated other Bayesian methods under both the informed input graph and the fully connected (uninformed) input graph where applicable (Fig 5C, 5D; S20 Table). As with baycn, sampling-based methods were run for 5 million iterations with 20% burn-in and 1,000 retained samples. baycn was computationally feasible under both settings and produced stable posterior probabilities. orderMCMC and partitionMCMC recovered all the key edges an informed input graph but were not practical under a fully connected input graph. BCDAG does not accept an informed graph as input, was feasible only under the uninformative graph but failed to recover nearly the key edges (Fig 5D). scanBMA does not accept input graphs and missed nearly half of the key edges. These results highlight a practical distinction between methods that require informative graph constraints for feasibility and baycn, which remains applicable under conservative, uninformative input graphs while quantifying uncertainty in edge recovery.

## Discussion and conclusion

We introduced baycn, a Bayesian approach to learning DAGs that represents a DAG by a vector of edge states. Specifically, our method departs from existing Bayesian methods in several key ways. First, we reformulate the graph prior at the level of edge states rather than node orderings, partitions, or entire DAGs (see Table 1). This representation enables direct probabilistic inference on edge direction and absence, simplifies prior specification, and allows posterior probabilities to be interpreted locally at the edge level, while naturally incorporating sparsity assumptions and biological knowledge through user-specified priors and input graphs. Second, our Metropolis-Hastings-like sampler enables efficient exploration of large, constrained graph spaces, yielding posterior edge probabilities that respect Markov equivalence when the data are informative. Our sampler also takes a pseudo-likelihood approach that removes potentially high-dimensional nuisance parameters. This enables efficient inference in settings where existing likelihood-based Bayesian approaches are computationally infeasible. Third, baycn accommodates mixed discrete and continuous data types that are common in genomics and supports causal network inference with instrumental variables to impose constraints on edge direction.

A caveat when interpreting the inferred network is that the direction of an edge indicates statistical dependence. With additional assumptions, the direction may also indicate the actual, causal mechanism. In the application of the eQTLs and their target genes, for example, we imposed the constraint that an edge between an eQTL and a gene always points to the latter. This is consistent with the biological principle that in general, DNA regulates RNA, but not the other way around. With this constraint, other directed edges may suggest regulatory relationships. For example, the subgraph [rs11305802 → PNP → TMEM55B] in Fig 4 suggests that the eQTL rs11305803 likely regulates gene TMEM55B through gene PNP.

Like all DAG-based causal models, our approach relies on the causal Markov assumption [5] for causal inference, which states that each variable is conditionally independent of its non-descendants given its direct parents in the graph. Violations of this assumption may arise when unmeasured confounders induce dependence between non-adjacent nodes, for example when a latent variable *U* induces association between $T_{1}$ and $T_{3}$ through $T_{1}\leftarrow U\to T_{3}$, beyond what is implied by the directed path $T_{1}\to T_{2}\to T_{3}$. In practice, we mitigate this issue by explicitly incorporating observed proxies for confounding into the network when available. In Case study A, for example, we identify major sources of confounding using PCs derived from genome-wide expression data and include these PCs as additional nodes in the inferred graph, thereby allowing such confounding to be represented directly in the graph rather than being absorbed into spurious edges.

The graphs analyzed here are still relatively small; extending baycn to networks with hundreds of nodes remains future work. Nonetheless, the features summarized above give baycn expanded capabilities relative to existing Bayesian methods, allowing it to perform reliable inference in situations that pose challenges for existing approaches.

## Supporting information

S1 Text Details of Theorem 1. (PDF)

S1 Fig The true graph and true probabilities for each edge in topology GN4. Orange edges have Markov equivalent edges and cannot be deterministically inferred.
(PDF)

S2 FigThe Markov equivalence class of topology GN4.The edges in orange show all possible combinations of edge directions of the Markov equivalence class. Edge 1 is oriented $T_{1}\to T_{2}$ in one of the three graphs, giving a proportion of 0.33 for edge state 0. Edge 2 is oriented $T_{2}\to T_{4}$ in two of the three graphs, giving a proportion of 0.66 for state 0.(PDF)

S3 Fig The true graph and true probabilities for each edge in topology GN5. The edges in orange can change direction while remaining in the Markov equivalence class of the true graph – as long as another v structure is not created.
(PDF)

S4 FigThe Markov equivalence class of topology GN5.The edges in orange show all possible combinations of edge directions of the Markov equivalence class. In two of the four graphs edge 1 is oriented $T_{1}\to T_{2}$, giving a proportion of 0.5 for edge state 0. In three of the four graphs edge 2 is oriented $T_{1}\to T_{3}$, giving a proportion of 0.75 for edge state 0. Similarly, edge 3 is oriented $T_{2}\to T_{4}$ in three of the four graphs, giving a proportion of 0.75 for edge state 0. These orange edges cannot be deterministically inferred.(PDF)

S5 Fig The true graph and probabilities for each edge in the multi-parent topology. (PDF)

S6 Fig The true graph and probabilities for each edge in topology GN11. The edges in orange can change direction while remaining in the Markov equivalence class of the true graph – as long as another v structure is not created. These orange edges cannot be deterministically inferred.
(PDF)

S7 Fig The true graph and probabilities for each edge in topology GN8. The edges in orange can change direction while remaining in the Markov equivalence class of the true graph – as long as another v structure is not created. These orange edges cannot be deterministically inferred.
(PDF)

S8 FigTrace plots of sampled graphs and their log pseudo-likelihoods from running baycn on simulate datasets.The figure uses a 3×3 layout, with rows representing graph topologies and columns representing sample sizes. Each cell contains a pair of trace plots: top is the trace plot of the log pseudo-likelihoods, and bottom is the trace plot of the sample graphs. Each unique configuration of a graph is represented by a distinct integer, converted from the vector of edge states. Short runs are shown for mixing diagnostics while keeping memory usage manageable and visualization feasible. For each topology-sample size combination, a single signal strength is randomly selected (e.g., GN4 at sample sizes 100, 200, and 600 with signal strengths 1.00, 0.2, and 0.5, respectively).(PDF)

S9 Fig The black edges (true edges) were used to simulate the data and the red edges (false edges) were added to the true adjacency matrix as input to baycn. (PDF)

S10 Fig The flowchart of using baycn for data analysis. (PDF)

S11 Fig Trace plots of sampled graphs and their log pseudo-likelihoods from three runs on the GEUVADIS eQTL-gene set Q8. Each run had 50,000 iterations with 20% burn-in and 40,000 retained samples. In each pair of plots: top is the trace plot of the log pseudo-likelihoods, and bottom is the trace plot of the sample graphs. Each unique configuration of a graph is represented by a distinct integer, converted from the vector of edge states. Short runs are shown for mixing diagnostics while keeping memory usage manageable and visualization feasible.
(PDF)

S12 Fig Inference of the GEUVADIS eQTL-gene set Q20, which does not have associated PCs. (A) Inferred graph with posterior probabilities. Numbers in parenthesis next to the edges indicate the posterior probability for the direction shown. (B) Correlation heatmap.
(PDF)

S13 Fig Inference of the GEUVADIS eQTL-gene set Q21 with associated PCs. (A) Inferred graph with posterior probabilities. Numbers in parenthesis next to the edges indicate the posterior probability for the direction shown. (B) Correlation heatmap.
(PDF)

S14 Fig Inference of the GEUVADIS eQTL-gene set Q23 with associated PCs. (A) Inferred graph with posterior probabilities. Numbers in parenthesis next to the edges indicate the posterior probability for the direction shown. (B) Correlation heatmap.
(PDF)

S15 Fig Inference of the GEUVADIS eQTL-gene set Q237 with associated PCs. (A) Inferred graph with posterior probabilities. Numbers in parenthesis next to the edges indicate the posterior probability for the direction shown. (B) Correlation heatmap.
(PDF)

S16 Fig Inference of the GEUVADIS eQTL-gene set Q50 with associated PCs. (A) Inferred graph with posterior probabilities. Numbers in parenthesis next to the edges indicate the posterior probability for the direction shown. (B) Correlation heatmap.
(PDF)

S17 Fig Inference of the GEUVADIS eQTL-gene set Q62, which does not have associated PCs. (A) Inferred graph with posterior probabilities. Numbers in parenthesis next to the edges indicate the posterior probability for the direction shown. (B) Correlation heatmap.
(PDF)

S18 Fig The informed input graph for the Drosophila data. We first used the graph output by MRPC then added additional edges between Meso&SM and the transcription factors Mef2 and Tin and between VM and the transcription factors Bin and Bap.
(PDF)

S19 Fig Trace plots of sampled graphs and their log pseudo-likelihoods from three runs on the drosophila data. Each run had 50,000 iterations with 20% burn-in and 40,000 retained samples. In each pair of plots: top is the trace plot of the log pseudo-likelihoods, and bottom is the trace plot of the sample graphs. Each unique configuration of a graph is represented by a distinct integer, converted from the vector of edge states. Short runs are shown for mixing diagnostics while keeping memory usage manageable and visualization feasible.
(PDF)

S20 Fig Inferred graph from baycn for the Drosophila data under a fully connected input graph with the tissue types treated as instrumental variables. To avoid confusion when interpreting directed edges in time-series data, we use a dot in place of an arrow. Except for bidirected edges, only the inferred direction with the corresponding posterior probability is shown. Posterior probabilities were averaged over three independent runs of 5 million iterations. Shades of the TFs reflect the timing of the TF binding.
(PDF)

S1 Table Performance of baycn measured in MSE_1_ on all the graphs used in simulation studies. Features of the graphs in Fig 1, such as the number of edges and v-structures, are listed. The mean and standard deviation of MSE_1_ (on three states of each edge), sample size *N*, and signal strength *β* are also listed. For each simulation scenario we generated 25 independent datasets and ran baycn once on each dataset.
(PDF)

S2 Table The mean and standard deviation of the edge-wise MSE for each edge in topology M1. We used the datasets previously simulated for M1 and included one false edge with the true edges in the input to baycn. We ran baycn with three different priors on edge states for each dataset. The rows in red represent false edges.
(PDF)

S3 Table The mean and standard deviation of the edge-wise MSE for each edge in topology M2. We used the datasets previously simulated for M2 and included one false edge with the true edges in the input to baycn. We ran baycn with three different priors on edge states for each dataset. The rows in red represent false edges.
(PDF)

S4 Table The mean and standard deviation of the edge-wise MSE for each edge in topology GN4. We used the datasets previously simulated for GN4 and included one false edge with the true edges in the input to baycn. We ran baycn with three different priors on edge states for each dataset. The rows in red represent false edges.
(PDF)

S5 Table The mean and standard deviation of the edge-wise MSE for each edge in topology GN11. We used the datasets previously simulated for GN11 and included two false edges with the true edges in the input to baycn. We ran baycn with three different priors on edge states for each dataset. The rows in red represent false edges.
(PDF)

S6 Table The mean and standard deviation of precision, power and MSE_2_ for GN4, GN11 and GN8 when a fully connected graph was used as input. We simulated 25 datasets for each combination of topology, *N*, and *β*, and ran each method once per dataset.
(XLSX)

S7 Table Posterior probabilities from baycn on the GEUVADIS eQTL-gene set Q8 with five associated PCs included in the network as confounding variables. A fully connected graph (excluding the edges between PC nodes) was used as the input to baycn. The rows highlighted in yellow indicate the edges between the nodes of interest.
(PDF)

S8 Table Posterior probabilities from order MCMC on the GEUVADIS eQTL-gene set Q8 with associated PCs. A fully connected graph (excluding the edges between PC nodes) was used as the input.
(PDF)

S9 Table Posterior probabilities from partition MCMC on the GEUVADIS eQTL-gene set Q8 with associated PCs. A fully connected graph (excluding the edges between PC nodes) was used as the input.
(PDF)

S10 Table Posterior probabilities from BCDAG on the GEUVADIS eQTL-gene set Q8 with associated PCs. A fully connected graph was used as the input, since BCDAG does not allow edge selection.
(PDF)

S11 Table Posterior probabilities from scanBMA on the GEUVADIS eQTL-gene set Q8 with associated PCs. A fully connected graph (excluding the edges between PC nodes) was used as the input.
(PDF)

S12 Table Posterior probabilities from baycn on the GEUVADIS eQTL-gene set Q8 without the associated PCs. A fully connected graph was used as the input.
(PDF)

S13 Table Posterior probabilities from baycn on the GEUVADIS eQTL-gene set Q20. A fully connected graph was used as the input.
(PDF)

S14 Table Posterior probabilities from baycn for the GEUVADIS eQTL-gene set Q21 with two PCs are included in the network as confounding variables. A fully connected graph was used as the input. The rows highlighted in yellow indicate the edges between the nodes of interest.
(PDF)

S15 Table Posterior probabilities from baycn on the GEUVADIS eQTL-gene set Q23 with two PCs included in the network as confounding variables. A fully connected graph was used as input. The rows highlighted in yellow indicate the edges between the nodes of interest.
(PDF)

S16 Table Posterior probabilities from baycn on the GEUVADIS eQTL-gene set Q37 with one PC included in the network as a confounding variable. A fully connected graph was used as input. The rows highlighted in yellow indicate the edges between the nodes of interest.
(PDF)

S17 Table Posterior probabilities from baycn on the GEUVADIS eQTL-gene set Q50 with two PCs included in the network as confounding variables. A fully connected graph was used as input. The rows highlighted in yellow indicate the edges between the nodes of interest.
(PDF)

S18 Table Posterior probabilities from baycn on the GEUVADIS eQTL-gene set Q62. A fully connected graph was used as input.
(PDF)

S19 Table Posterior probabilities of edge states from baycn on the Drosophila data. Sheet 1 shows the results under the informed input graph, and Sheet 2 under the fully connect input graph.
(XLSX)

S20 Table Posterior probabilities from order MCMC, partition MCMC, BCDAG and scanBMA on the Drosophila data. Each sheet shows the results from a method.
(XLSX)

## References

[1] Friedman N. Inferring cellular networks using probabilistic graphical models. Science. 2004;303(5659):799–805. doi: 10.1126/science.1094068 14764868

[2] Rau A, Jaffrézic F, Foulley J-L, Doerge RW. Reverse engineering gene regulatory networks using approximate Bayesian computation. Stat Comput. 2011;22(6):1257–71. doi: 10.1007/s11222-011-9309-1

[3] Gomez SM, Noble WS, Rzhetsky A. Learning to predict protein-protein interactions from protein sequences. Bioinformatics. 2003;19(15):1875–81. doi: 10.1093/bioinformatics/btg352 14555619

[4] Zhang QC, Petrey D, Deng L, Qiang L, Shi Y, Thu CA, et al. Structure-based prediction of protein-protein interactions on a genome-wide scale. Nature. 2012;490(7421):556–60. doi: 10.1038/nature11503 23023127 PMC3482288

[5] Spirtes P, Glymour CN, Scheines R. Causation, Prediction, and Search. MIT Press; 2000.

[6] Guyon I, Janzing D, Schölkopf B. Causality: objectives and assessment. JMLR Workshop and Conference Proceedings. 2010. p. 1–38.

[7] Dawid AP. Beware of the DAG!. JMLR Workshop and Conference Proceedings. 2010. p. 59–86.

[8] McGuire AL, Gabriel S, Tishkoff SA, Wonkam A, Chakravarti A, Furlong EEM, et al. The road ahead in genetics and genomics. Nat Rev Genet. 2020;21(10):581–96. doi: 10.1038/s41576-020-0272-6 32839576 PMC7444682

[9] Davidson EH. Emerging properties of animal gene regulatory networks. Nature. 2010;468(7326):911–20. doi: 10.1038/nature09645 21164479 PMC3967874

[10] Guelzim N, Bottani S, Bourgine P, Képès F. Topological and causal structure of the yeast transcriptional regulatory network. Nat Genet. 2002;31(1):60–3. doi: 10.1038/ng873 11967534

[11] Schwaller L, Robin S, Stumpf M. Closed-form Bayesian inference of graphical model structures by averaging over trees. Journal de la Société Française de Statistique. 2019;160(2):1–23.

[12] Leday GGR, Richardson S. Fast Bayesian inference in large Gaussian graphical models. Biometrics. 2019;75(4):1288–98. doi: 10.1111/biom.13064 31009060 PMC6916355

[13] Yeung KY, Dombek KM, Lo K, Mittler JE, Zhu J, Schadt EE, et al. Construction of regulatory networks using expression time-series data of a genotyped population. Proc Natl Acad Sci U S A. 2011;108(48):19436–41. doi: 10.1073/pnas.1116442108 22084118 PMC3228453

[14] Lo K, Raftery AE, Dombek KM, Zhu J, Schadt EE, Bumgarner RE, et al. Integrating external biological knowledge in the construction of regulatory networks from time-series expression data. BMC Syst Biol. 2012;6:101. doi: 10.1186/1752-0509-6-101 22898396 PMC3465231

[15] Young WC, Raftery AE, Yeung KY. Fast Bayesian inference for gene regulatory networks using ScanBMA. BMC Syst Biol. 2014;8:47. doi: 10.1186/1752-0509-8-47 24742092 PMC4006459

[16] Fronczuk M, Raftery AE, Yeung KY. CyNetworkBMA: a Cytoscape app for inferring gene regulatory networks. Source Code Biol Med. 2015;10:11. doi: 10.1186/s13029-015-0043-5 26566394 PMC4642660

[17] Hung L-H, Shi K, Wu M, Young WC, Raftery AE, Yeung KY. fastBMA: scalable network inference and transitive reduction. Gigascience. 2017;6(10):1–10. doi: 10.1093/gigascience/gix078 29020744 PMC5632288

[18] Madigan D, York J, Allard D. Bayesian graphical models for discrete data. International Statistical Review / Revue Internationale de Statistique. 1995;63(2):215. doi: 10.2307/1403615

[19] Friedman N, Koller D. Being Bayesian about network structure. A Bayesian approach to structure discovery in Bayesian networks. Machine Learning. 2003;50(1–2):95–125. doi: 10.1023/a:1020249912095

[20] Giudici P, Castelo R. Improving Markov chain Monte Carlo model search for data mining. Machine Learning. 2003;50(1–2):127–58. doi: 10.1023/a:1020202028934

[21] Grzegorczyk M, Husmeier D. Improving the structure MCMC sampler for Bayesian networks by introducing a new edge reversal move. Mach Learn. 2008;71(2–3):265–305. doi: 10.1007/s10994-008-5057-7

[22] He Y, Jia J, Yu B. Reversible MCMC on Markov equivalence classes of sparse directed acyclic graphs. Ann Statist. 2013;41(4). doi: 10.1214/13-aos1125

[23] Mohammadi A, Wit EC. Bayesian Structure Learning in Sparse Gaussian Graphical Models. Bayesian Anal. 2015;10(1). doi: 10.1214/14-ba889

[24] Goudie RJB, Mukherjee S. A Gibbs Sampler for Learning DAGs. J Mach Learn Res. 2016;17(30):1–39. 28331463 PMC5358773

[25] Su C, Borsuk ME. Improving structure MCMC for Bayesian networks through Markov blanket resampling. The Journal of Machine Learning Research. 2016;17(1):4042–61.

[26] Kuipers J, Moffa G. Partition MCMC for Inference on Acyclic Digraphs. Journal of the American Statistical Association. 2017;112(517):282–99. doi: 10.1080/01621459.2015.1133426

[27] Castelletti F, Consonni G. Objective Bayes model selection of Gaussian interventional essential graphs for the identification of signaling pathways. Ann Appl Stat. 2019;13(4). doi: 10.1214/19-aoas1275

[28] Rezaei Tabar V, Zareifard H, Salimi S, Plewczynski D. An empirical Bayes approach for learning directed acyclic graph using MCMC algorithm. Statistical Analysis. 2019;12(5):394–403. doi: 10.1002/sam.11430

[29] Viinikka J, Koivisto M. Layering-MCMC for Structure Learning in Bayesian Networks. In: Proceedings of the Conference on Uncertainty in Artificial Intelligence, 2020. 839–48.

[30] Castelletti F, Consonni G. Discovering Causal Structures in Bayesian Gaussian Directed Acyclic Graph Models. Journal of the Royal Statistical Society Series A: Statistics in Society. 2020;183(4):1727–45. doi: 10.1111/rssa.12550

[31] Castelletti F, Mascaro A. Structural learning and estimation of joint causal effects among network-dependent variables. Stat Methods Appl. 2021;30(5):1289–314. doi: 10.1007/s10260-021-00579-1

[32] Kuipers J, Suter P, Moffa G. Efficient Sampling and Structure Learning of Bayesian Networks. Journal of Computational and Graphical Statistics. 2022;31(3):639–50. doi: 10.1080/10618600.2021.2020127

[33] Castelletti F, Peluso S. Network Structure Learning Under Uncertain Interventions. Journal of the American Statistical Association. 2022;118(543):2117–28. doi: 10.1080/01621459.2022.2037430

[34] Castelletti F, Mascaro A. Bcdag: An r package for bayesian structure and causal learning of gaussian dags. arXiv. 2022.

[35] Badsha MB, Martin EA, Fu AQ. MRPC: An R Package for Inference of Causal Graphs. Front Genet. 2021;12:651812. doi: 10.3389/fgene.2021.651812 33995486 PMC8120292

[36] Kalisch M, Mächler M, Colombo D, Maathuis MH, Bühlmann P. Causal Inference Using Graphical Models with theRPackagepcalg. J Stat Soft. 2012;47(11). doi: 10.18637/jss.v047.i11

[37] Scutari M. Learning Bayesian Networks with thebnlearnRPackage. J Stat Soft. 2010;35(3). doi: 10.18637/jss.v035.i03

[38] Badsha MB, Fu AQ. Learning Causal Biological Networks With the Principle of Mendelian Randomization. Front Genet. 2019;10:460. doi: 10.3389/fgene.2019.00460 31164902 PMC6536645

[39] Kvamme J, Badsha MB, Martin EA, Wu J, Wang X, Fu AQ. Causal network inference of cis- and trans-gene regulation of expression quantitative trait loci across human tissues. Genetics. 2025;230(2):iyaf064. doi: 10.1093/genetics/iyaf064 40179003 PMC12135172

[40] VanderWeele TJ, Tchetgen Tchetgen EJ, Cornelis M, Kraft P. Methodological challenges in mendelian randomization. Epidemiology. 2014;25(3):427–35. doi: 10.1097/EDE.0000000000000081 24681576 PMC3981897

[41] Davey Smith G, Hemani G. Mendelian randomization: genetic anchors for causal inference in epidemiological studies. Hum Mol Genet. 2014;23(R1):R89-98. doi: 10.1093/hmg/ddu328 25064373 PMC4170722

[42] Emdin CA, Khera AV, Kathiresan S. Mendelian Randomization. JAMA. 2017;318(19):1925–6. doi: 10.1001/jama.2017.17219 29164242

[43] Verma T, Pearl J. In: Proceedings of the Sixth Annual Conference on Uncertainty in Artificial Intelligence, 1990. 255–70.

[44] Ventura L, Racugno W. Pseudo-Likelihoods for Bayesian Inference. Topics on Methodological and Applied Statistical Inference. Springer International Publishing. 2016. p. 205–20. 10.1007/978-3-319-44093-4_19

[45] Severini TA. Likelihood methods in statistics. Oxford University Press. 2000.

[46] Severini TA. On the relationship between Bayesian and non-Bayesian elimination of nuisance parameters. Statistica Sinica. 1999;:713–24.

[47] Tarjan R. Depth-First Search and Linear Graph Algorithms. SIAM J Comput. 1972;1(2):146–60. doi: 10.1137/0201010

[48] Cheung VG, Spielman RS. Genetics of human gene expression: mapping DNA variants that influence gene expression. Nat Rev Genet. 2009;10(9):595–604. doi: 10.1038/nrg2630 19636342 PMC2989458

[49] Lappalainen T, Sammeth M, Friedländer MR, ’t Hoen PAC, Monlong J, Rivas MA, et al. Transcriptome and genome sequencing uncovers functional variation in humans. Nature. 2013;501(7468):506–11. doi: 10.1038/nature12531 24037378 PMC3918453

[50] Sanderson E, Glymour MM, Holmes MV, Kang H, Morrison J, Munafò MR, et al. Mendelian randomization. Nat Rev Methods Primers. 2022;2:6. doi: 10.1038/s43586-021-00092-5 37325194 PMC7614635

[51] Stegle O, Parts L, Piipari M, Winn J, Durbin R. Using probabilistic estimation of expression residuals (PEER) to obtain increased power and interpretability of gene expression analyses. Nat Protoc. 2012;7(3):500–7. doi: 10.1038/nprot.2011.457 22343431 PMC3398141

[52] Holm S. A simple sequentially rejective multiple test procedure. Scandinavian Journal of Statistics. 1979;:65–70.

[53] Villar D, Flicek P, Odom DT. Evolution of transcription factor binding in metazoans - mechanisms and functional implications. Nat Rev Genet. 2014;15(4):221–33. doi: 10.1038/nrg3481 24590227 PMC4175440

[54] Zinzen RP, Girardot C, Gagneur J, Braun M, Furlong EEM. Combinatorial binding predicts spatio-temporal cis-regulatory activity. Nature. 2009;462(7269):65–70. doi: 10.1038/nature08531 19890324

[55] Stojnic R, Fu AQ, Adryan B. A graphical modelling approach to the dissection of highly correlated transcription factor binding site profiles. PLoS Comput Biol. 2012;8(11):e1002725. doi: 10.1371/journal.pcbi.1002725 23144600 PMC3493460

[56] Zeitlinger J, Zinzen RP, Stark A, Kellis M, Zhang H, Young RA, et al. Whole-genome ChIP-chip analysis of Dorsal, Twist, and Snail suggests integration of diverse patterning processes in the Drosophila embryo. Genes Dev. 2007;21(4):385–90. doi: 10.1101/gad.1509607 17322397 PMC1804326

[57] Sandmann T, Girardot C, Brehme M, Tongprasit W, Stolc V, Furlong EEM. A core transcriptional network for early mesoderm development in Drosophila melanogaster. Genes Dev. 2007;21(4):436–49. doi: 10.1101/gad.1509007 17322403 PMC1804332

[58] Azpiazu N, Frasch M. tinman and bagpipe: two homeo box genes that determine cell fates in the dorsal mesoderm of Drosophila. Genes Dev. 1993;7(7B):1325–40. doi: 10.1101/gad.7.7b.1325 8101173

[59] Lockwood WK, Bodmer R. The patterns of wingless, decapentaplegic, and tinman position the Drosophila heart. Mech Dev. 2002;114(1–2):13–26. doi: 10.1016/s0925-4773(02)00044-8 12175486

[60] Lilly B, Galewsky S, Firulli AB, Schulz RA, Olson EN. D-MEF2: a MADS box transcription factor expressed in differentiating mesoderm and muscle cell lineages during Drosophila embryogenesis. Proc Natl Acad Sci U S A. 1994;91(12):5662–6. doi: 10.1073/pnas.91.12.5662 8202544 PMC44056

[61] Bour BA, O’Brien MA, Lockwood WL, Goldstein ES, Bodmer R, Taghert PH, et al. Drosophila MEF2, a transcription factor that is essential for myogenesis. Genes Dev. 1995;9(6):730–41. doi: 10.1101/gad.9.6.730 7729689

[62] Gunthorpe D, Beatty KE, Taylor MV. Different levels, but not different isoforms, of the Drosophila transcription factor DMEF2 affect distinct aspects of muscle differentiation. Dev Biol. 1999;215(1):130–45. doi: 10.1006/dbio.1999.9449 10525355

[63] Jakobsen JS, Braun M, Astorga J, Gustafson EH, Sandmann T, Karzynski M, et al. Temporal ChIP-on-chip reveals Biniou as a universal regulator of the visceral muscle transcriptional network. Genes Dev. 2007;21(19):2448–60. doi: 10.1101/gad.437607 17908931 PMC1993875

[64] Zaffran S, Küchler A, Lee HH, Frasch M. biniou (FoxF), a central component in a regulatory network controlling visceral mesoderm development and midgut morphogenesis in Drosophila. Genes Dev. 2001;15(21):2900–15. doi: 10.1101/gad.917101 11691840 PMC312807

