# Supplementary material for: Approximate Bayesian inference of directed acyclic graphs in biology with flexible priors on edge states
Source: PLoS Comput Biol. 2026 Mar 16;22(3):e1014039. doi: 10.1371/journal.pcbi.1014039 (PMC13046286; doi:10.1371/journal.pcbi.1014039)
Supplement: S1 Text — (PDF) [file pcbi.1014039.s001.pdf]

Approximate Bayesian inference of directed acyclic graphs in biology with flexible priors on edge states

Supplementary Notes

Note A: Calculating the transition probability in our sampling algorithm

Once a suitable proposal is generated, our sampling algorithm next calculates the acceptance probability for the proposed graph relative to the current one. Although the (repeated) removal of directed cycles enters the calculation of the transition probability between the current and proposed graph, the probabilities involving the cycles are in the end canceled in the calculation of the acceptance ratio. Let $\mathbf{D}$ be a vector of indices of the edges that *differ* between the current graph $\mathbf{S}$ and proposed graph $\mathbf{S}'$ and $\mathbf{C}$ be an integer vector where the element c_j indicates the number of edges that *can* change state for the edge denoted by d_j (see examples in Section). These two vectors have the same length, denoted by h. The probability of moving from $\mathbf{S}$ to $\mathbf{S}'$, i.e., $\Pr(\mathbf{S} \to \mathbf{S}')$, is the product of the probabilities of changes at individual edges in $\mathbf{D}$, and each of these probabilities further consists of two probabilities: the probability that an edge in the graph is chosen to change states, which is $1/c_j$, and the probability of edge d_j changing from its current state S_{d_j} to the state S'_{d_j}, denoted by $\Pr(S_{d_j} \to S'_{d_j})$. Therefore,

$$\Pr(\mathbf{S} \to \mathbf{S}') = \prod_{j=1}^{h} \frac{1}{c_j} \Pr(S_{d_j} \to S'_{d_j}) = \prod_{j=1}^{h} \frac{1}{c_j} \prod_{j=1}^{h} \Pr(S_{d_j} \to S'_{d_j}). \tag{1}$$

We prove that the transition probabilities do not depend on the path taken from the current graph to the proposed graph (the process of introducing and removing directed cycles) but only on the edges that have different states between the two graphs.

Theorem 1 *When calculating the acceptance probability, the transition probabilities between the current graph* $\mathbf{S}$ *and proposed graph* $\mathbf{S}'$, $\Pr(S \to S')$ *and* $\Pr(S' \to S)$, *depend only on the edges whose states are different between the two graphs.*

Proof Recall that $\mathbf{S}$ is a vector of edge states representing the current graph $\mathcal{G}$ and $\mathbf{S}'$ is a vector of edge states representing the proposal graph $\mathcal{G}'$. Let $\mathbf{D}$ be a vector of indices of the edges that *differ* between the current graph $\mathbf{S}$ and proposed graph $\mathbf{S}'$ and $\mathbf{C}$ be an integer vector where the element c_j represents the number of edges that *can* change state for the edge represented by d_j. These two vectors have the same length, denoted by h.

We will consider two cases: without and with potential directed cycles in the graph.

Case 1 Without potential directed cycles the probability of moving from the current graph to the proposed graph is

$$\begin{aligned}\Pr(\mathbf{S} \to \mathbf{S}') &= \prod_{j=1}^{h} \frac{1}{c_j} \Pr(S_{d_j} \to S'_{d_j}) \\ &= \prod_{j=1}^{h} \frac{1}{c_j} \prod_{j=1}^{h} \Pr(S_{d_j} \to S'_{d_j}).\end{aligned} \tag{2}$$

We can use the same procedure of deriving the equation for moving back to the current graph from the proposed graph. Therefore, the probability can be broken down in the same way when moving backwards:

$$\Pr(\mathbf{S}' \to \mathbf{S}) = \prod_{j=1}^{h} \frac{1}{c_j} \prod_{j=1}^{h} \Pr(S'_{d_j} \to S_{d_j}). \tag{3}$$

Since there are no potential directed cycles in the network the value c_j will always be m which is the number of edges in the network. Therefore, $\prod_{j=1}^{h} \frac{1}{c_j} = \prod_{j=1}^{h} \frac{1}{m}$ whether going from $\mathbf{S} \to \mathbf{S}'$ or $\mathbf{S}' \to \mathbf{S}$ and will cancel when calculating the acceptance probability, leaving $\prod_{j=1}^{h} \Pr(S_{d_j} \to S'_{d_j})$ and $\prod_{j=1}^{h} \Pr(S'_{d_j} \to S_{d_j})$.

Case 2 With potential directed cycles there can be multiple paths when moving from the current graph to the proposed graph. Let $\mathbf{C}^k$ be a vector where each c_j^k is the number of edges that can change state in path k when moving from $\mathbf{S}$ to $\mathbf{S}'$ and $\mathbf{C}^{k'}$ be a vector where each $c_j^{k'}$ is the number of edges that can change state in path k when moving from $\mathbf{S}'$ to $\mathbf{S}$. Using Equation (2) the transition probability of moving from the current graph to the proposed graph when there are multiple paths becomes

$$\Pr(\mathbf{S} \to \mathbf{S}') = \sum_{k=1}^{K} \prod_{j=1}^{h} \frac{1}{c_j^k} \prod_{j=1}^{h} \Pr(S_{d_j} \to S'_{d_j}) = \prod_{j=1}^{h} \Pr(S_{d_j} \to S'_{d_j}) \sum_{k=1}^{K} \prod_{j=1}^{h} \frac{1}{c_j^k}. \tag{4}$$

Similarly, the transition probability when there are multiple paths of moving back to the current graph from the proposed graph is

$$\Pr(\mathbf{S}' \to \mathbf{S}) = \sum_{k=1}^{K} \prod_{j=1}^{h} \frac{1}{c_j^{k'}} \prod_{j=1}^{h} \Pr(S'_{d_j} \to S_{d_j}) = \prod_{j=1}^{h} \Pr(S'_{d_j} \to S_{d_j}) \sum_{k=1}^{K} \prod_{j=1}^{h} \frac{1}{c_j^{k'}}. \tag{5}$$

In Equations (4) and (5) the summation over K represents the different paths (Section) to get from one graph to another and the last equality holds because the edges that are different between $\mathbf{S}$ and $\mathbf{S}'$ do not depend on the path k.

For each path k

$$\begin{aligned}
\mathbf{C}^k &= (c_1^k, c_2^k, c_3^k, \ldots, c_h^k) && (6)\\
&= (\underbrace{c_1^k, c_2^k, \ldots, c_j^k}_{\text{create cycle(s)}}, \underbrace{c_{j+1}^k, \ldots, c_h^k}_{\text{remove cycle(s)}}) && (7)\\
&= (\underbrace{m, \ldots, m}_{j}, c_{j+1}^k, \ldots, c_h^k). && (8)
\end{aligned}$$

The first j elements can create one or more directed cycles. The remaining $h-j$ elements then remove the cycle(s) that were introduced in the network and their values are equal to the number of edges that make up the directed cycle that is being removed. The cycles that are created and removed in any path k from $\mathbf{S}$ to $\mathbf{S}'$ can also be created and removed when moving from $\mathbf{S}'$ to $\mathbf{S}$. Therefore, the equations for moving from the proposed graph to the current graph will be the same as Equations (6) - (8) except for the $'$ symbol indicating that we are moving backwards:

$$\begin{aligned}
\mathbf{C}^{k'} &= (c_1^{k'}, c_2^{k'}, c_3^{k'}, \ldots, c_h^{k'}) && (9)\\
&= (\underbrace{c_1^{k'}, c_2^{k'}, \ldots, c_j^{k'}}_{\text{create cycle(s)}}, \underbrace{c_{j+1}^{k'}, \ldots, c_h^{k'}}_{\text{remove cycle(s)}}) && (10)\\
&= (\underbrace{m, \ldots, m}_{j}, c_{j+1}^{k'}, \ldots, c_h^{k'}), && (11)
\end{aligned}$$

and

$$\sum_{k=1}^{K} \prod_{j=1}^{h} \frac{1}{c_j^k} = \sum_{k=1}^{K} \prod_{j=1}^{h} \frac{1}{c_j^{k'}}. \tag{12}$$

The terms in Equation (12) will cancel when calculating the acceptance probability and we will be left with $\prod_{j=1}^{h} \Pr(S_{d_j} \to S'_{d_j})$ and $\prod_{j=1}^{h} \Pr(S'_{d_j} \to S_{d_j})$. ■

See Note B for examples.

Note B: Examples of Theorem 1

Example 1 – one directed cycle

Consider the current graph in Figure 1a with states $\mathbf{S} = (0, 0, 0, 1, 1)$ and the proposed graph in Figure 1c with states $\mathbf{S}' = (0, 1, 2, 1, 1)$. Edges #2 and #3 have different states between $\mathbf{S}$ and $\mathbf{S}'$ therefore $\mathbf{D} = 2, 3$. There are two different

paths to move from $\mathbf{S}$ to $\mathbf{S}'$. In path 1 there are two steps: i) edge #2 changes direction which creates a directed cycle between nodes T_1, T_2, and T_3 (Figure 1b) and ii) the directed cycle is removed by edge #3 changing from state 0 to 2. In path 2 edges #2 and #3 change states in one step. If the prior on edge states is $p_0 = 0.05$, $p_1 = 0.05$ and $p_2 = 0.9$ then the probabilities for the two paths are

$$\text{path 1: } \Pr(S_{d_2} \to S'_{d_2}) = \frac{0.05}{0.95}, \ \Pr(S_{d_3} \to S'_{d_3}) = \frac{0.9}{0.95}, \ \mathbf{C}^1 = 5, 3$$

and

$$\text{path 2: } \Pr(S_{d_2} \to S'_{d_2}) = \frac{0.05}{0.95}, \ \Pr(S_{d_3} \to S'_{d_3}) = \frac{0.9}{0.95}, \ \mathbf{C}^2 = 5, 5.$$

Combining the probabilities from each path we obtain the transition probability of moving to the proposed graph:

$$\Pr(\mathbf{S} \to \mathbf{S}') = \frac{1}{5}\frac{1}{3}\frac{0.05}{0.95}\frac{0.9}{0.95} + \frac{1}{5}\frac{1}{5}\frac{0.05}{0.95}\frac{0.9}{0.95} = \Big(\frac{1}{5}\frac{1}{3} + \Big(\frac{1}{5}\Big)^2\Big)\frac{0.05}{0.95}\frac{0.9}{0.95}. \tag{13}$$

Any directed cycle created when moving from $\mathbf{S}$ to $\mathbf{S}'$ needs to be created when moving from $\mathbf{S}'$ to $\mathbf{S}$. Therefore, when moving from $\mathbf{S}'$ to $\mathbf{S}$ there are also two paths. Path 1 is made up of two steps: i) edge #3 changes from state 2 to 0 creating a cycle between nodes T_1, T_2, and T_3 and ii) the cycle is removed by changing edge #2 from state 1 to 0. For path 2 edges #2 and #3 both change states in one step. The probabilities for the paths are

$$\text{path 1: } \Pr(S'_{d_2} \to S_{d_2}) = \frac{0.05}{0.95}, \ \Pr(S'_{d_3} \to S_{d_3}) = \frac{0.05}{0.1}, \ \mathbf{C}^{1'} = 5, 3$$

and

$$\text{path 2: } \Pr(S'_{d_2} \to S_{d_2}) = \frac{0.05}{0.95}, \ \Pr(S'_{d_3} \to S_{d_3}) = \frac{0.05}{0.1}, \ \mathbf{C}^{2'} = 5, 5.$$

The transition probability of moving back to the current graph is

$$\Pr(\mathbf{S}' \to \mathbf{S}) = \frac{1}{5}\frac{1}{3}\frac{0.05}{0.95}\frac{0.05}{0.1} + \frac{1}{5}\frac{1}{5}\frac{0.05}{0.95}\frac{0.05}{0.1} = \Big(\frac{1}{5}\frac{1}{3} + \Big(\frac{1}{5}\Big)^2\Big)\frac{0.05}{0.95}\frac{0.05}{0.1}. \tag{14}$$

The term $\frac{1}{5}\frac{1}{3} + \left(\frac{1}{5}\right)^2$ in Equations (13) and (14) cancels out when calculating the acceptance probability, α, and we are left with the probability of moving between the states that differ between the current graph (Figure 1a) and the proposed graph (Figure 1c). More generally, we can apply the same procedure to traverse the paths between any two graphs.

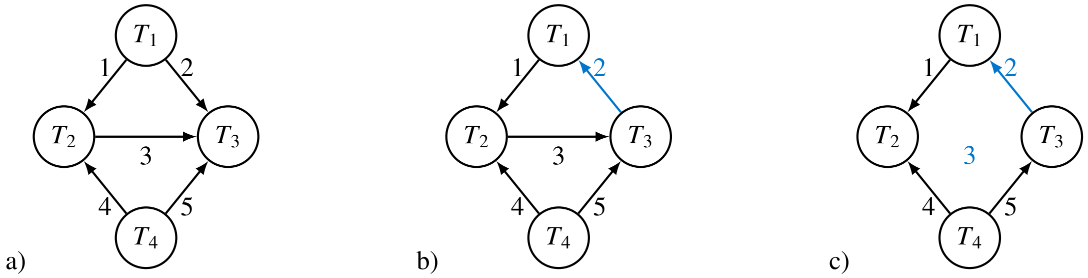

Figure 1. The graphs for example 1. a) The current graph. b) An intermediate graph between the current graph and the proposed graph where a directed cycle has been introduced into the network. c) The proposed graph.

Example 2 – multiple directed cycles

We show a second more complex example below. If we start with the graph in Figure 2a with states $\mathbf{S} = (0, 0, 0, 1, 1)$ and the proposed graph in Figure 2d with states $\mathbf{S}' = (2, 1, 1, 1, 0)$ there are four edges with different states between the two graphs and $\mathbf{D} = 1, 2, 3, 5$. There are three different paths to move from $\mathbf{S}$ to $\mathbf{S}'$. The steps in path 1 are: i) edges #2 and #5 change directions creating two directed cycles, the first cycle is between nodes T_1, T_2, and T_3 and the second cycle is between nodes T_2, T_3, and T_4 (Figure 2b), ii) edge #1 changes from state 0 to 2 removing the first

cycle (Figure 2c), and iii) edge #3 changes direction which removes the second cycle (Figure 2d). The steps in path 2 are: i) edges #1, #2, and #5 all change states creating one directed cycle between nodes T_2, T_3, and T_4 (Figure 2c) and ii) edge #3 changes direction removing the cycle. In path 3 edges #1, #2, #3, and #5 all change states in one step. If the prior on edge states is $p_0 = 0.05$, $p_1 = 0.05$, and $p_2 = 0.9$ then the probabilities for the three paths are:

$$\text{path 1: } \Pr(S_{d_1} \to S'_{d_1}) = \frac{0.9}{0.95}, \Pr(S_{d_2} \to S'_{d_2}) = \frac{0.05}{0.95}, \Pr(S_{d_3} \to S'_{d_3}) = \frac{0.05}{0.95}, \Pr(S_{d_5} \to S'_{d_5}) = \frac{0.05}{0.95}, \mathbf{C}^1 = 5, 5, 3, 3,$$

$$\text{path 2: } \Pr(S_{d_1} \to S'_{d_1}) = \frac{0.9}{0.95}, \Pr(S_{d_2} \to S'_{d_2}) = \frac{0.05}{0.95}, \Pr(S_{d_3} \to S'_{d_3}) = \frac{0.05}{0.95}, \Pr(S_{d_5} \to S'_{d_5}) = \frac{0.05}{0.95}, \mathbf{C}^2 = 5, 5, 5, 3,$$

and

$$\text{path 3: } \Pr(S_{d_1} \to S'_{d_1}) = \frac{0.9}{0.95}, \Pr(S_{d_2} \to S'_{d_2}) = \frac{0.05}{0.95}, \Pr(S_{d_3} \to S'_{d_3}) = \frac{0.05}{0.95}, \Pr(S_{d_5} \to S'_{d_5}) = \frac{0.05}{0.95}, \mathbf{C}^3 = 5, 5, 5, 5.$$

Therefore, the transition probability of moving to the proposed graph is

$$\begin{aligned}
\Pr(\mathbf{S} \to \mathbf{S}') &= \frac{1}{5}\frac{1}{5}\frac{1}{3}\frac{1}{3}\frac{0.9}{0.95}\frac{0.05}{0.95}\frac{0.05}{0.95}\frac{0.05}{0.95} + \frac{1}{5}\frac{1}{5}\frac{1}{5}\frac{1}{3}\frac{0.9}{0.95}\frac{0.05}{0.95}\frac{0.05}{0.95}\frac{0.05}{0.95} + \frac{1}{5}\frac{1}{5}\frac{1}{5}\frac{1}{5}\frac{0.9}{0.95}\frac{0.05}{0.95}\frac{0.05}{0.95}\frac{0.05}{0.95} \\
&= \left(\left(\frac{1}{5}\right)^2\left(\frac{1}{3}\right)^2 + \left(\frac{1}{5}\right)^3\frac{1}{3} + \left(\frac{1}{5}\right)^4\right)\frac{0.9}{0.95}\frac{0.05}{0.95}\frac{0.05}{0.95}\frac{0.05}{0.95}. \qquad (15)
\end{aligned}$$

As in example 1 any directed cycle that is created when moving from $\mathbf{S}$ to $\mathbf{S}'$ needs to also be created when moving from $\mathbf{S}'$ to $\mathbf{S}$. There are also three different paths to move back to $\mathbf{S}$ from $\mathbf{S}'$. In path 1 the steps are: i) edges #1 and #3 change states creating two directed cycles the first cycle is between nodes T_1, T_2, and T_3 and the second cycle is between nodes T_2, T_3, and T_4, ii) edge #2 changes direction removing the first cycle, and iii) edge #5 changes direction removing the second directed cycle. Path 2 has two steps i) edges #1, #2 and #3 all change state creating a directed cycle between nodes T_2, T_3, and T_4 and ii) edge #5 changes direction removing the cycle. In path 3 there is only one step where edges #1, #2, #3, and #5 all change states. The probabilities for these paths are

$$\text{path 1: } \Pr(S'_{d_1} \to S_{d_1}) = \frac{0.05}{0.1}, \Pr(S'_{d_2} \to S_{d_2}) = \frac{0.05}{0.95}, \Pr(S'_{d_3} \to S_{d_3}) = \frac{0.05}{0.95}, \Pr(S'_{d_5} \to S_{d_5}) = \frac{0.05}{0.95}, \mathbf{C}^{1'} = 5, 5, 3, 3,$$

$$\text{path 2: } \Pr(S'_{d_1} \to S_{d_1}) = \frac{0.05}{0.1}, \Pr(S'_{d_2} \to S_{d_2}) = \frac{0.05}{0.95}, \Pr(S'_{d_3} \to S_{d_3}) = \frac{0.05}{0.95}, \Pr(S'_{d_5} \to S_{d_5}) = \frac{0.05}{0.95}, \mathbf{C}^{2'} = 5, 5, 5, 3,$$

and

$$\text{path 3: } \Pr(S'_{d_1} \to S_{d_1}) = \frac{0.05}{0.1}, \Pr(S'_{d_2} \to S_{d_2}) = \frac{0.05}{0.95}, \Pr(S'_{d_3} \to S_{d_3}) = \frac{0.05}{0.95}, \Pr(S'_{d_5} \to S_{d_5}) = \frac{0.05}{0.95}, \mathbf{C}^{3'} = 5, 5, 5, 5.$$

Therefore, the transition probability of moving back to the current graph is

$$\begin{aligned}
\Pr(\mathbf{S}' \to \mathbf{S}) &= \frac{1}{5}\frac{1}{5}\frac{1}{3}\frac{1}{3}\frac{0.05}{0.1}\frac{0.05}{0.95}\frac{0.05}{0.95}\frac{0.05}{0.95} + \frac{1}{5}\frac{1}{5}\frac{1}{5}\frac{1}{3}\frac{0.05}{0.1}\frac{0.05}{0.95}\frac{0.05}{0.95}\frac{0.05}{0.95} + \frac{1}{5}\frac{1}{5}\frac{1}{5}\frac{1}{5}\frac{0.05}{0.1}\frac{0.05}{0.95}\frac{0.05}{0.95}\frac{0.05}{0.95} \\
&= \left(\left(\frac{1}{5}\right)^2\left(\frac{1}{3}\right)^2 + \left(\frac{1}{5}\right)^3\frac{1}{3} + \left(\frac{1}{5}\right)^4\right)\frac{0.05}{0.1}\frac{0.05}{0.95}\frac{0.05}{0.95}\frac{0.05}{0.95}. \qquad (16)
\end{aligned}$$

The term $\left(\frac{1}{5}\right)^2\left(\frac{1}{3}\right)^2+\left(\frac{1}{5}\right)^3\frac{1}{3}+\left(\frac{1}{5}\right)^4$ in Equations (15) and (16) cancels out when calculating the acceptance probability, α, and we are left with the probability of moving between the states that differ between the current graph (Figure 2a) and the proposed graph (Figure 2d). More generally, we can apply the same procedure to traverse the paths between any two graphs.

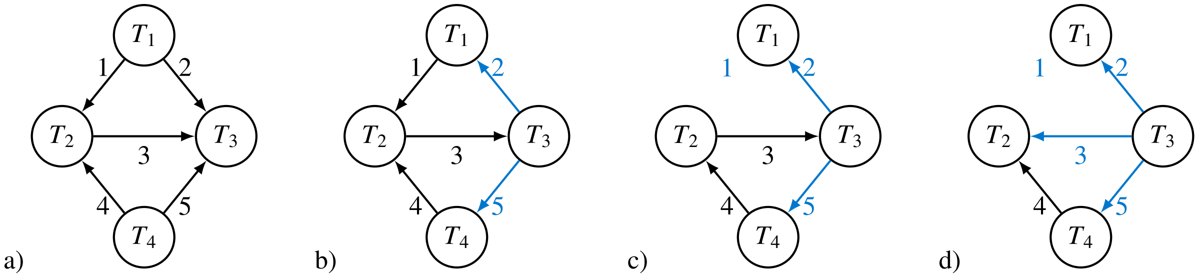

Figure 2. a) The current graph. b) An intermediate graph between the current graph and the proposed graph where two directed cycles have been introduced into the graph. c) An intermediate graph where one of the directed cycles has been removed. d) The proposed graph.
