# Supplementary material for: Approximate Bayesian inference of directed acyclic graphs in biology with flexible priors on edge states
Source: PLoS Comput Biol. 2026 Mar 16;22(3):e1014039. doi: 10.1371/journal.pcbi.1014039 (PMC13046286; doi:10.1371/journal.pcbi.1014039)
Supplement: S1 Fig — Orange edges have Markov equivalent edges and cannot be deterministically inferred. (PDF) [file pcbi.1014039.s002.pdf]

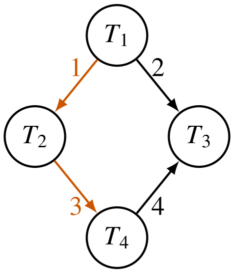

	True Probability		
Edge	0	1	2
1	0.33	0.66	0
2	1	0	0
3	0.66	0.33	0
4	0	1	0

S1 Fig. The true graph and probabilities for each edge in topology GN4. Orange edges have Markov equivalent edges and cannot be deterministically inferred.
