# Supplementary material for: Approximate Bayesian inference of directed acyclic graphs in biology with flexible priors on edge states
Source: PLoS Comput Biol. 2026 Mar 16;22(3):e1014039. doi: 10.1371/journal.pcbi.1014039 (PMC13046286; doi:10.1371/journal.pcbi.1014039)
Supplement: S2 Fig — The edges in orange show all possible combinations of edge directions of the Markov equivalence class. Edge 1 is oriented T1→T2 in one of the three graphs, giving a proportion of 0.33 for edge state 0. Edge 2 is oriented T2→T4 in two of the three graphs, giving a proportion of 0.66 for state 0. (PDF) [file pcbi.1014039.s003.pdf]

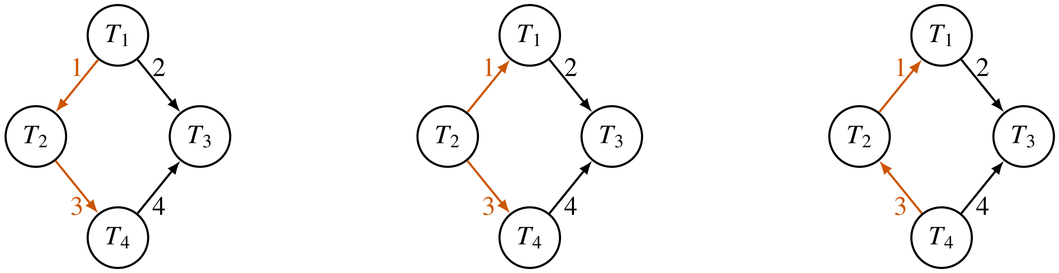

S2 Fig. The Markov equivalence class of topology GN4. The edges in orange show all possible combinations of edge directions of the Markov equivalence class. Edge 1 is oriented $T_1 \rightarrow T_2$ in one of the three graphs, giving a proportion of 0.33 for edge state 0. Edge 2 is oriented $T_2 \rightarrow T_4$ in two of the three graphs, giving a proportion of 0.66 for state 0.
