# Supplementary material for: Approximate Bayesian inference of directed acyclic graphs in biology with flexible priors on edge states
Source: PLoS Comput Biol. 2026 Mar 16;22(3):e1014039. doi: 10.1371/journal.pcbi.1014039 (PMC13046286; doi:10.1371/journal.pcbi.1014039)
Supplement: S3 Fig — The edges in orange can change direction while remaining in the Markov equivalence class of the true graph – as long as another v structure is not created. (PDF) [file pcbi.1014039.s004.pdf]

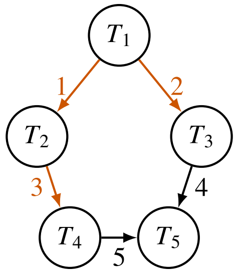

Edge	True Probability 0	1	2
1	0.5	0.5	0
2	0.75	0.25	0
3	0.75	0.25	0
4	1	0	0
5	1	0	0

S3 Fig. The true graph and probabilities for each edge in topology GN5. The edges in orange can change direction while remaining in the Markov equivalence class of the true graph – as long as another v-structure is not created. These orange edges cannot be deterministically inferred.
