# Supplementary material for: Approximate Bayesian inference of directed acyclic graphs in biology with flexible priors on edge states
Source: PLoS Comput Biol. 2026 Mar 16;22(3):e1014039. doi: 10.1371/journal.pcbi.1014039 (PMC13046286; doi:10.1371/journal.pcbi.1014039)
Supplement: S4 Fig — The edges in orange show all possible combinations of edge directions of the Markov equivalence class. In two of the four graphs edge 1 is oriented T1→T2, giving a proportion of 0.5 for edge state 0. In three of the four graphs edge 2 is oriented T1→T3, giving a proportion of 0.75 for edge state 0. Similarly, edge 3 is oriented T2→T4 in three of the four graphs, giving a proportion of 0.75 for edge state 0. These orange edges cannot be deterministically inferred. (PDF) [file pcbi.1014039.s005.pdf]

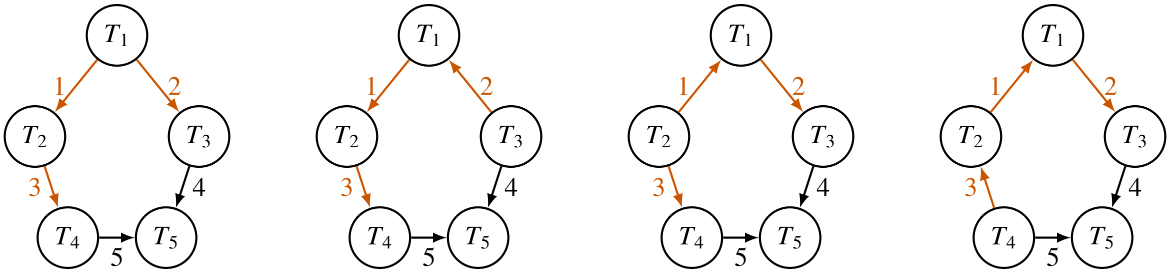

S4 Fig. The Markov equivalence class of topology GN5. The edges in orange show all possible combinations of edge directions of the Markov equivalence class. In two of the four graphs edge 1 is oriented $T_1 \rightarrow T_2$, giving a proportion of 0.5 for edge state 0. In three of the four graphs edge 2 is oriented $T_1 \rightarrow T_3$, giving a proportion of 0.75 for edge state 0. Similarly, edge 3 is oriented $T_2 \rightarrow T_4$ in three of the four graphs, giving a proportion of 0.75 for edge state 0. These orange edges cannot be deterministically inferred.
