# Supplementary figures and images for: Approximate Bayesian inference of directed acyclic graphs in biology with flexible priors on edge states
Source: PLoS Comput Biol. 2026 Mar 16;22(3):e1014039. doi: 10.1371/journal.pcbi.1014039 (PMC13046286; doi:10.1371/journal.pcbi.1014039)

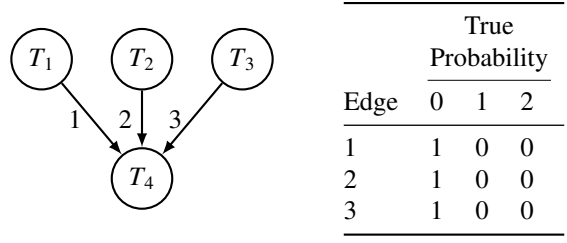

S5 Fig. The true graph and probabilities for each edge in the multi-parent topology.

Supplement: S5 Fig — (PDF) [file pcbi.1014039.s006.pdf]

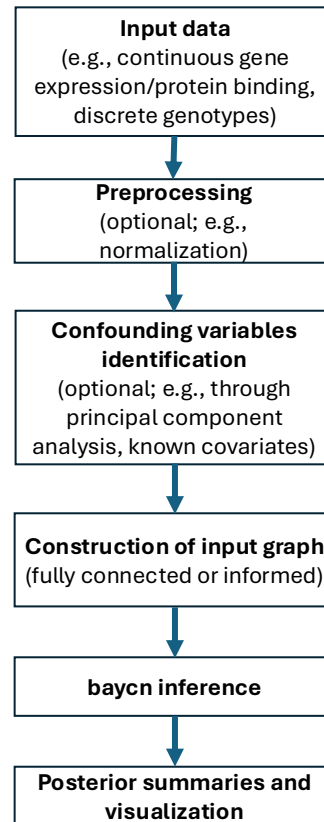

S10 Fig. The flowchart of using baycn for data analysis.

Supplement: S10 Fig — (PDF) [file pcbi.1014039.s011.pdf]
