# Supplementary material for: Approximate Bayesian inference of directed acyclic graphs in biology with flexible priors on edge states
Source: PLoS Comput Biol. 2026 Mar 16;22(3):e1014039. doi: 10.1371/journal.pcbi.1014039 (PMC13046286; doi:10.1371/journal.pcbi.1014039)
Supplement: S6 Fig — The edges in orange can change direction while remaining in the Markov equivalence class of the true graph – as long as another v structure is not created. These orange edges cannot be deterministically inferred. (PDF) [file pcbi.1014039.s007.pdf]

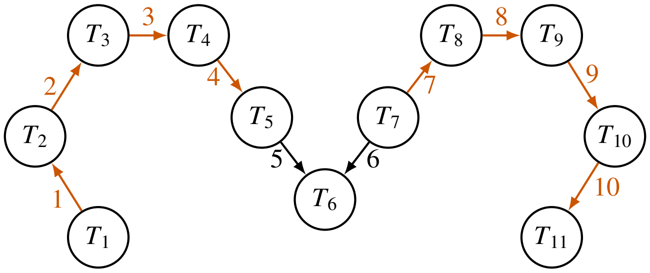

	True Probability		
Edge	0	1	2
1	0.2	0.8	0
2	0.4	0.6	0
3	0.6	0.4	0
4	0.8	0.2	0
5	1	0	0
6	0	1	0
7	0.2	0.8	0
8	0.4	0.6	0
9	0.6	0.4	0
10	0.8	0.2	0

S6 Fig. The true graph and probabilities for each edge in topology GN11. The edges in orange can change direction while remaining in the Markov equivalence class of the true graph – as long as another v-structure is not created. These orange edges cannot be deterministically inferred.
