# Supplementary material for: Approximate Bayesian inference of directed acyclic graphs in biology with flexible priors on edge states
Source: PLoS Comput Biol. 2026 Mar 16;22(3):e1014039. doi: 10.1371/journal.pcbi.1014039 (PMC13046286; doi:10.1371/journal.pcbi.1014039)
Supplement: S7 Fig — The edges in orange can change direction while remaining in the Markov equivalence class of the true graph – as long as another v structure is not created. These orange edges cannot be deterministically inferred. (PDF) [file pcbi.1014039.s008.pdf]

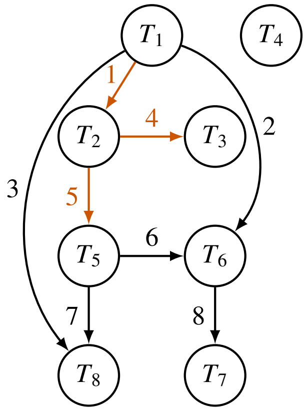


| | True Probability | | |
|---|---|---|---|
| Edge | 0 | 1 | 2 |
| 1 | 0.25 | 0.75 | 0 |
| 2 | 1 | 0 | 0 |
| 3 | 1 | 0 | 0 |
| 4 | 0.75 | 0.25 | 0 |
| 5 | 0.75 | 0.25 | 0 |
| 6 | 1 | 0 | 0 |
| 7 | 1 | 0 | 0 |
| 8 | 1 | 0 | 0 |

S7 Fig. The true graph and probabilities for each edge in topology GN8. The edges in orange can change direction while remaining in the Markov equivalence class of the true graph – as long as another v-structure is not created. These orange edges cannot be deterministically inferred.
