# Supplementary material for: Approximate Bayesian inference of directed acyclic graphs in biology with flexible priors on edge states
Source: PLoS Comput Biol. 2026 Mar 16;22(3):e1014039. doi: 10.1371/journal.pcbi.1014039 (PMC13046286; doi:10.1371/journal.pcbi.1014039)
Supplement: S8 Fig — The figure uses a 3×3 layout, with rows representing graph topologies and columns representing sample sizes. Each cell contains a pair of trace plots: top is the trace plot of the log pseudo-likelihoods, and bottom is the trace plot of the sample graphs. Each unique configuration of a graph is represented by a distinct integer, converted from the vector of edge states. Short runs are shown for mixing diagnostics while keeping memory usage manageable and visualization feasible. For each topology-sample size combination, a single signal strength is randomly selected (e.g., GN4 at sample sizes 100, 200, and 600 with signal strengths 1.00, 0.2, and 0.5, respectively). (PDF) [file pcbi.1014039.s009.pdf]

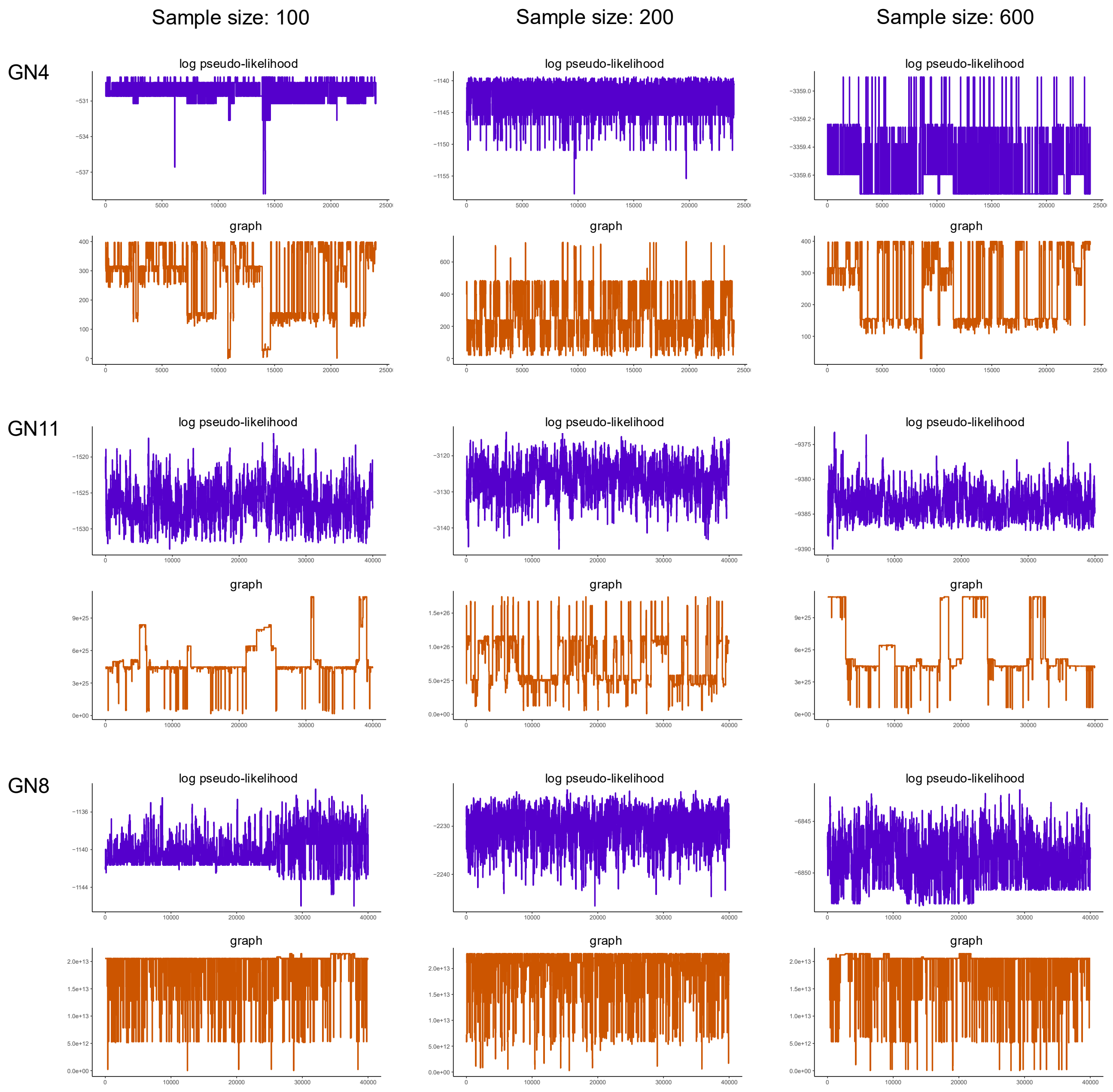

S8 Fig. Trace plots of sampled graphs and their log pseudo-likelihoods from running baycn on simulate datasets. The figure uses a 3×3 layout, with rows representing graph topologies and columns representing sample sizes. Each cell contains a pair of trace plots: top is the trace plot of the log pseudo-likelihoods, and bottom is the trace plot of the sample graphs. Each unique configuration of a graph is represented by a distinct integer, converted from the vector of edge states. Short runs are shown for mixing diagnostics while keeping memory usage manageable and visualization feasible. For each topology-sample size combination, a single signal strength is randomly selected (e.g., GN4 at sample sizes 100, 200, and 600 with signal strengths 1.00, 0.2, and 0.5, respectively).
