# Supplementary material for: Approximate Bayesian inference of directed acyclic graphs in biology with flexible priors on edge states
Source: PLoS Comput Biol. 2026 Mar 16;22(3):e1014039. doi: 10.1371/journal.pcbi.1014039 (PMC13046286; doi:10.1371/journal.pcbi.1014039)
Supplement: S9 Fig — (PDF) [file pcbi.1014039.s010.pdf]

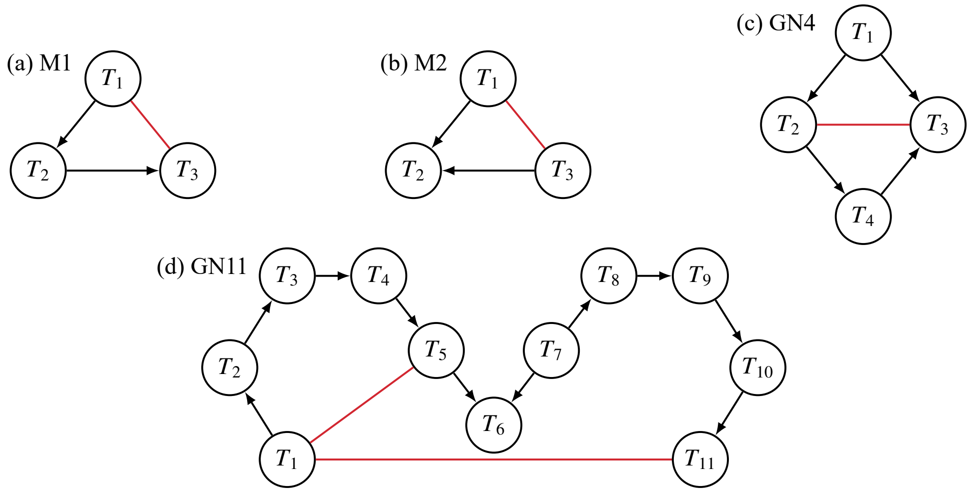


S9 Fig. The black edges (true edges) were used to simulate the data and the red edges (false edges) were added to the true adjacency matrix as input to baycn.
