# Supplementary material for: Approximate Bayesian inference of directed acyclic graphs in biology with flexible priors on edge states
Source: PLoS Comput Biol. 2026 Mar 16;22(3):e1014039. doi: 10.1371/journal.pcbi.1014039 (PMC13046286; doi:10.1371/journal.pcbi.1014039)
Supplement: S12 Fig — (A) Inferred graph with posterior probabilities. Numbers in parenthesis next to the edges indicate the posterior probability for the direction shown. (B) Correlation heatmap. (PDF) [file pcbi.1014039.s013.pdf]

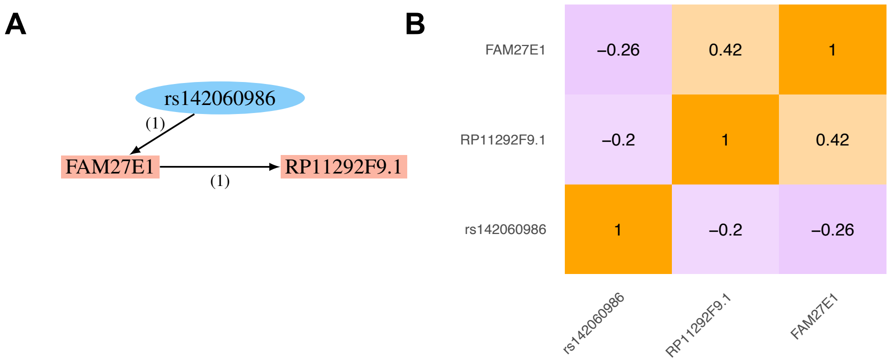

S12 Fig. Inference of the GEUVADIS eQTL-gene set Q20, which does not have associated PCs. (A) Inferred graph with posterior probabilities. Numbers in parenthesis next to the edges indicate the posterior probability for the direction shown. (B) Correlation heatmap.
