# Supplementary material for: Approximate Bayesian inference of directed acyclic graphs in biology with flexible priors on edge states
Source: PLoS Comput Biol. 2026 Mar 16;22(3):e1014039. doi: 10.1371/journal.pcbi.1014039 (PMC13046286; doi:10.1371/journal.pcbi.1014039)
Supplement: S13 Fig — (A) Inferred graph with posterior probabilities. Numbers in parenthesis next to the edges indicate the posterior probability for the direction shown. (B) Correlation heatmap. (PDF) [file pcbi.1014039.s014.pdf]

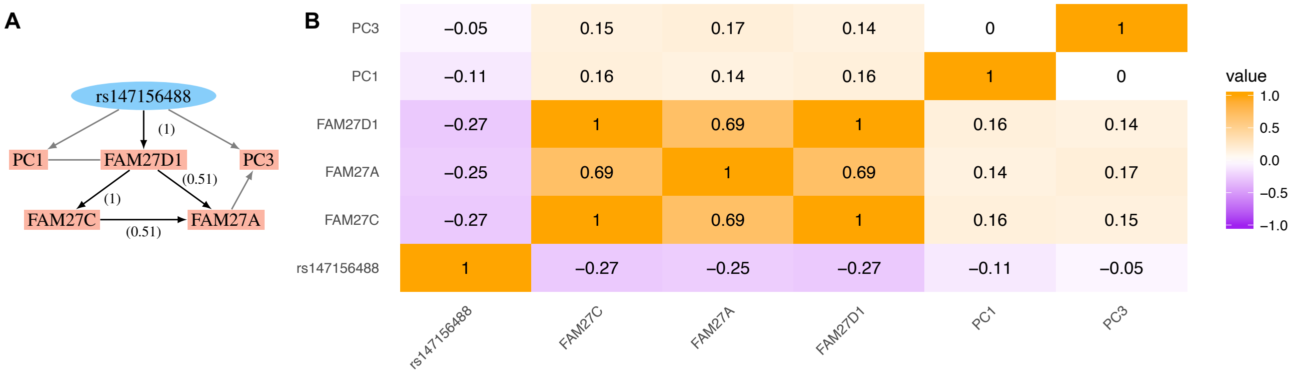

S13 Fig. Inference of the GEUVADIS eQTL-gene set Q21 with associated PCs. (A) Inferred graph with posterior probabilities. Numbers in parenthesis next to the edges indicate the posterior probability for the direction shown. (B) Correlation heatmap.
