# Supplementary material for: Approximate Bayesian inference of directed acyclic graphs in biology with flexible priors on edge states
Source: PLoS Comput Biol. 2026 Mar 16;22(3):e1014039. doi: 10.1371/journal.pcbi.1014039 (PMC13046286; doi:10.1371/journal.pcbi.1014039)
Supplement: S18 Fig — We first used the graph output by MRPC then added additional edges between Meso&SM and the transcription factors Mef2 and Tin and between VM and the transcription factors Bin and Bap. (PDF) [file pcbi.1014039.s019.pdf]

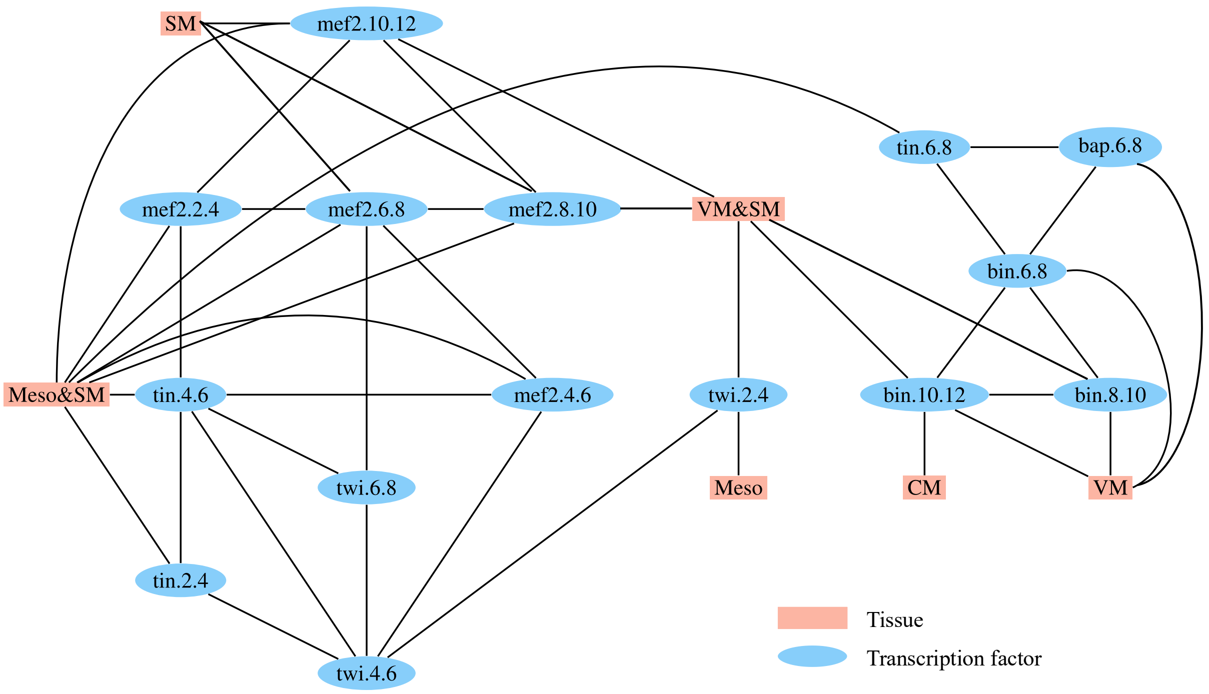

S18 Fig. The informed input graph for the Drosophila data. We first used the graph output by MRPC then added additional edges between Meso&SM and the transcription factors Mef2 and Tin and between VM and the transcription factors Bin and Bap.
