# Supplementary material for: Approximate Bayesian inference of directed acyclic graphs in biology with flexible priors on edge states
Source: PLoS Comput Biol. 2026 Mar 16;22(3):e1014039. doi: 10.1371/journal.pcbi.1014039 (PMC13046286; doi:10.1371/journal.pcbi.1014039)
Supplement: S19 Fig — Each run had 50,000 iterations with 20% burn-in and 40,000 retained samples. In each pair of plots: top is the trace plot of the log pseudo-likelihoods, and bottom is the trace plot of the sample graphs. Each unique configuration of a graph is represented by a distinct integer, converted from the vector of edge states. Short runs are shown for mixing diagnostics while keeping memory usage manageable and visualization feasible. (PDF) [file pcbi.1014039.s020.pdf]

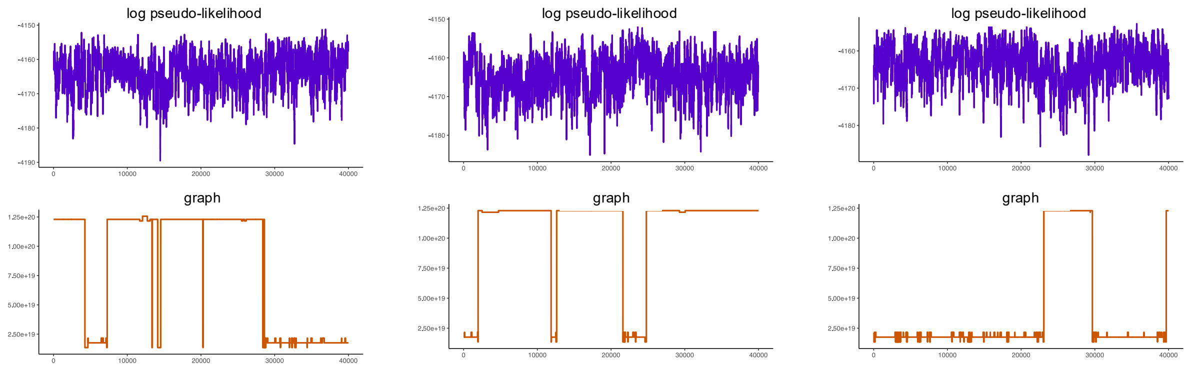

S19 Fig. Trace plots of sampled graphs and their log pseudo-likelihoods from three runs on the drosophila data. Each run had 50,000 iterations with 20% burn-in and 40,000 retained samples. In each pair of plots: top is the trace plot of the log pseudo-likelihoods, and bottom is the trace plot of the sample graphs. Each unique configuration of a graph is represented by a distinct integer, converted from the vector of edge states. Short runs are shown for mixing diagnostics while keeping memory usage manageable and visualization feasible.
