# Supplementary material for: Approximate Bayesian inference of directed acyclic graphs in biology with flexible priors on edge states
Source: PLoS Comput Biol. 2026 Mar 16;22(3):e1014039. doi: 10.1371/journal.pcbi.1014039 (PMC13046286; doi:10.1371/journal.pcbi.1014039)
Supplement: S20 Fig — To avoid confusion when interpreting directed edges in time-series data, we use a dot in place of an arrow. Except for bidirected edges, only the inferred direction with the corresponding posterior probability is shown. Posterior probabilities were averaged over three independent runs of 5 million iterations. Shades of the TFs reflect the timing of the TF binding. (PDF) [file pcbi.1014039.s021.pdf]

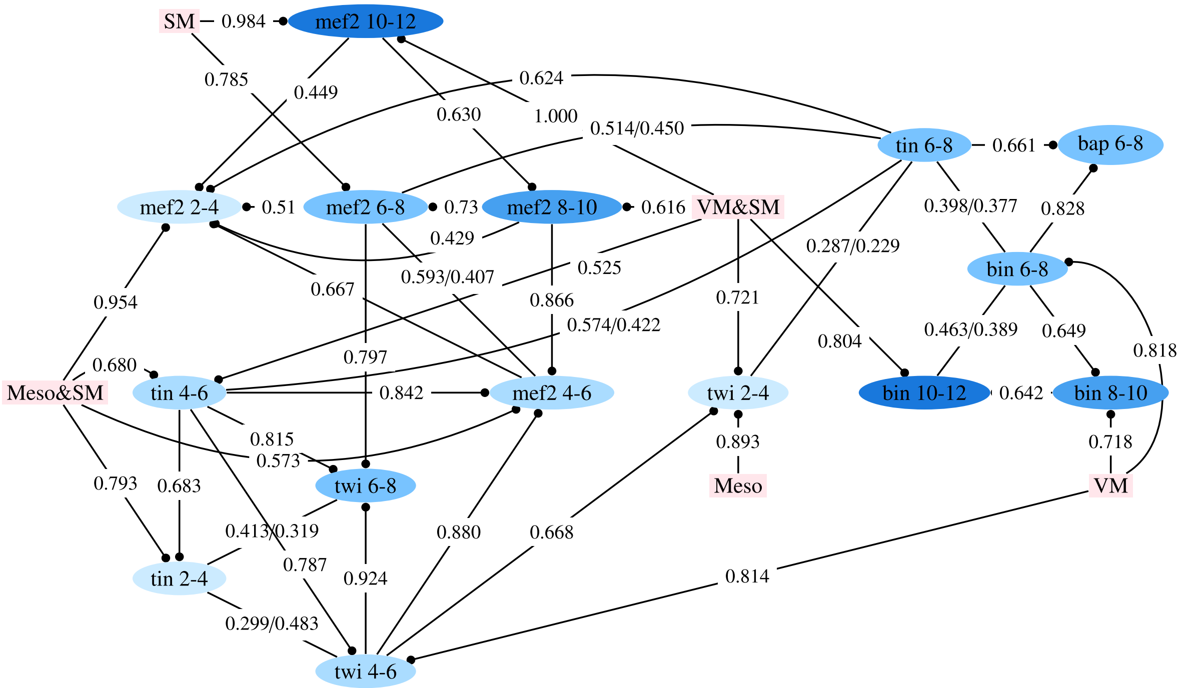

S20 Fig. Inferred graph from baycn for the Drosophila data under a fully connected input graph with the tissue types treated as instrumental variables. To avoid confusion when interpreting directed edges in time-series data, we use a dot in place of an arrow. Except for bidirected edges, only the inferred direction with the corresponding posterior probability is shown. Posterior probabilities were averaged over three independent runs of 5 million iterations. Shades of the TFs reflect the timing of the TF binding.
