# Supplementary material for: Approximate Bayesian inference of directed acyclic graphs in biology with flexible priors on edge states
Source: PLoS Comput Biol. 2026 Mar 16;22(3):e1014039. doi: 10.1371/journal.pcbi.1014039 (PMC13046286; doi:10.1371/journal.pcbi.1014039)
Supplement: S1 Table — Features of the graphs in Fig 1, such as the number of edges and v-structures, are listed. The mean and standard deviation of MSE1 (on three states of each edge), sample size N, and signal strength β are also listed. For each simulation scenario we generated 25 independent datasets and ran baycn once on each dataset. (PDF) [file pcbi.1014039.s022.pdf]

S1 Table. Performance of baycn measured in MSE_1 on all the graphs used in simulation studies. Features of the graphs in Figure 1 in the main text, such as the number of edges and v-structures, are listed. The mean and standard deviation of MSE_1 (on three states of each edge), sample size N, and signal strength β are also listed. For each simulation scenario we generated 25 independent data sets and ran baycn once on each data set.

				MSE_1					
				$\beta = 0.2$		$\beta = 0.5$		$\beta = 1$	
Topology	# edges	# v-structures	N	mean	sd	mean	sd	mean	sd
M1	2	0	100	0.1796	0.0949	0.0127	0.0353	0.0011	0.0016
			200	0.0734	0.074	0.0014	0.0015	0.0012	0.0013
			600	0.0237	0.0326	0.001	0.0009	0.0008	0.0007
M2	2	1	100	0.3384	0.1081	0.0909	0.0919	0	0
			200	0.2688	0.0881	0.0597	0.082	0	0
			600	0.1323	0.0735	0	0	0	0
GN4	4	1	100	0.2731	0.0711	0.0674	0.0417	0.01	0.0276
			200	0.167	0.0554	0.0755	0.0639	0.0046	0.0142
			600	0.0839	0.0259	0.0503	0.0602	0.0069	0.0182
GN5	5	1	100	0.2687	0.0785	0.0396	0.0465	0.0022	0.0023
			200	0.1562	0.049	0.0171	0.0235	0.002	0.0031
			600	0.0684	0.0338	0.0114	0.0483	0.002	0.0024
Mulit-parent	3	3	100	0.3361	0.0961	0.0486	0.0698	0	0
			200	0.237	0.0812	0.0032	0.0135	0	0
			600	0.1418	0.0625	0	0.0002	0	0
GN11	10	1	100	0.2266	0.0513	0.0353	0.0225	0.0042	0.0031
			200	0.139	0.0431	0.0121	0.0162	0.0066	0.0041
			600	0.0613	0.0197	0.0047	0.0043	0.0046	0.0039
GN8	8	2	100	0.2959	0.1517	0.0667	0.0666	0.0247	0.0526
			200	0.197	0.144	0.0692	0.0962	0.0186	0.0486
			600	0.1147	0.1073	0.0556	0.0858	0.0109	0.0467
