# Supplementary material for: Approximate Bayesian inference of directed acyclic graphs in biology with flexible priors on edge states
Source: PLoS Comput Biol. 2026 Mar 16;22(3):e1014039. doi: 10.1371/journal.pcbi.1014039 (PMC13046286; doi:10.1371/journal.pcbi.1014039)
Supplement: S3 Table — We used the datasets previously simulated for M2 and included one false edge with the true edges in the input to baycn. We ran baycn with three different priors on edge states for each dataset. The rows in red represent false edges. (PDF) [file pcbi.1014039.s024.pdf]

S3 Table. The mean and standard deviation of the edge-wise MSE for each edge in topology M2. We used the data sets previously simulated for M2 and included one false edge with the true edges in the input to baycn. We ran baycn with three different priors on edge states for each data set. The rows in red represent false edges.

		eMSE: Topology M2					
		Prior 1		Prior 2		Prior 3	
N	Edge	mean	sd	mean	sd	mean	sd
100	1	0.1366	0.0269	0.1197	0.0199	0.0224	0.026
	2	0.4035	0.0368	0.3285	0.0548	0.0656	0.0666
	3	0.1461	0.025	0.1241	0.0226	0.0232	0.028
200	1	0.1329	0.0262	0.1215	0.0222	0.0297	0.0354
	2	0.4111	0.0373	0.3474	0.0612	0.0863	0.0975
	3	0.1391	0.0227	0.1209	0.0245	0.0317	0.0396
600	1	0.1369	0.0207	0.1183	0.0221	0.0236	0.0164
	2	0.4062	0.0347	0.3362	0.0459	0.0647	0.0377
	3	0.1378	0.028	0.1186	0.0223	0.0224	0.0149
