# Supplementary material for: Approximate Bayesian inference of directed acyclic graphs in biology with flexible priors on edge states
Source: PLoS Comput Biol. 2026 Mar 16;22(3):e1014039. doi: 10.1371/journal.pcbi.1014039 (PMC13046286; doi:10.1371/journal.pcbi.1014039)
Supplement: S5 Table — We used the datasets previously simulated for GN11 and included two false edges with the true edges in the input to baycn. We ran baycn with three different priors on edge states for each dataset. The rows in red represent false edges. (PDF) [file pcbi.1014039.s026.pdf]

S5 Table. The mean and standard deviation of the edge-wise MSE for each edge in topology GN11. We used the data sets previously simulated for GN11 and included two false edges with the true edges in the input to baycn. We ran baycn with three different priors on edge states for each data set. The rows in red represent false edges.

		eMSE: Topology GN11					
		Prior 1		Prior 2		Prior 3	
N	Edge	mean	sd	mean	sd	mean	sd
100	1	0.0023	0.0035	0.003	0.0038	0.0046	0.006
	2	0.2976	0.1311	0.2084	0.1464	0.0386	0.1027
	3	0.2931	0.0658	0.2039	0.0851	0.0168	0.0239
	4	0.0018	0.0028	0.0035	0.0033	0.009	0.0104
	5	0.0029	0.0046	0.0037	0.0048	0.0087	0.0116
	6	0.0045	0.007	0.0043	0.0064	0.0038	0.0044
	7	0	0	0	0	0	0
	8	0	0	0	0	0	0
	9	0.0009	0.0011	0.0014	0.0023	0.0037	0.005
	10	0.0018	0.002	0.002	0.0025	0.0107	0.0153
	11	0.002	0.0019	0.0024	0.0029	0.0097	0.0132
	12	0.0016	0.0018	0.0016	0.0026	0.0044	0.0057
200	1	0.0048	0.0046	0.0033	0.0042	0.0029	0.0036
	2	0.286	0.1233	0.1938	0.1334	0.0338	0.0753
	3	0.3161	0.0676	0.2138	0.0676	0.0188	0.0152
	4	0.0032	0.0052	0.0028	0.0038	0.0076	0.0092
	5	0.0026	0.0043	0.0029	0.006	0.0069	0.0085
	6	0.0041	0.0056	0.0032	0.0056	0.0039	0.0052
	7	0	0	0	0	0	0
	8	0	0	0	0	0	0
	9	0.0006	0.0009	0.0004	0.0005	0.008	0.0137
	10	0.0013	0.0016	0.0007	0.0013	0.0115	0.0148
	11	0.0018	0.0021	0.0014	0.002	0.0093	0.0112
	12	0.0021	0.0025	0.0012	0.0018	0.0038	0.0041
600	1	0.0069	0.0069	0.0052	0.0058	0.0048	0.0056
	2	0.2735	0.0938	0.1783	0.0949	0.0147	0.0231
	3	0.3436	0.1118	0.2446	0.129	0.0557	0.1172
	4	0.0049	0.0062	0.0028	0.003	0.0108	0.0147
	5	0.0024	0.0029	0.0017	0.0021	0.0077	0.0112
	6	0.0027	0.003	0.0015	0.0015	0.0024	0.0023
	7	0	0	0	0	0	0
	8	0	0	0	0	0	0
	9	0.0006	0.0008	0.0009	0.0013	0.0031	0.0036
	10	0.0015	0.0018	0.0023	0.0033	0.0058	0.0069
	11	0.0017	0.0021	0.0029	0.0047	0.0066	0.0087
	12	0.0026	0.005	0.0022	0.0041	0.0046	0.0047
