# Supplementary material for: Approximate Bayesian inference of directed acyclic graphs in biology with flexible priors on edge states
Source: PLoS Comput Biol. 2026 Mar 16;22(3):e1014039. doi: 10.1371/journal.pcbi.1014039 (PMC13046286; doi:10.1371/journal.pcbi.1014039)
Supplement: S7 Table — A fully connected graph (excluding the edges between PC nodes) was used as the input to baycn. The rows highlighted in yellow indicate the edges between the nodes of interest. (PDF) [file pcbi.1014039.s028.pdf]

S7 Table. Posterior probabilities from baycn on the GEUVADIS eQTL-gene set Q8 with five associated PCs included in the network as confounding variables. A fully connected graph (excluding the edges between PC nodes, since PCs are independent of one another) was used as the input to baycn. The rows highlighted in yellow indicate the edges between the nodes of interest.

edge	forward	backward	absence
rs11305802-TMEM55B	0.208	0.000	0.792
rs11305802-RP11-203M5.8	0.727	0.000	0.273
rs11305802-PNP	1.000	0.000	0.000
rs11305802-PC1	0.274	0.000	0.726
rs11305802-PC2	0.140	0.000	0.860
rs11305802-PC6	0.267	0.000	0.733
rs11305802-PC7	0.144	0.000	0.856
rs11305802-PC9	0.374	0.000	0.626
TMEM55B-RP11-203M5.8	0.263	0.700	0.037
TMEM55B-PNP	0.311	0.686	0.004
TMEM55B-PC1	0.360	0.632	0.008
TMEM55B-PC2	0.142	0.458	0.400
TMEM55B-PC6	0.029	0.067	0.904
TMEM55B-PC7	0.040	0.051	0.908
TMEM55B-PC9	0.281	0.705	0.015
RP11-203M5.8-PNP	0.629	0.371	0.000
RP11-203M5.8-PC1	0.073	0.116	0.811
RP11-203M5.8-PC2	0.141	0.304	0.555
RP11-203M5.8-PC6	0.366	0.634	0.000
RP11-203M5.8-PC7	0.161	0.084	0.755
RP11-203M5.8-PC9	0.061	0.080	0.859
PNP-PC1	0.225	0.678	0.098
PNP-PC2	0.213	0.732	0.055
PNP-PC6	0.044	0.085	0.871
PNP-PC7	0.483	0.270	0.246
PNP-PC9	0.191	0.624	0.185
