# Supplementary material for: Approximate Bayesian inference of directed acyclic graphs in biology with flexible priors on edge states
Source: PLoS Comput Biol. 2026 Mar 16;22(3):e1014039. doi: 10.1371/journal.pcbi.1014039 (PMC13046286; doi:10.1371/journal.pcbi.1014039)
Supplement: S9 Table — A fully connected graph (excluding the edges between PC nodes) was used as the input. (PDF) [file pcbi.1014039.s030.pdf]

S9 Table. Posterior probabilities from partition MCMC on the GEUVADIS eQTL-gene set Q8 with associated PCs. A fully connected graph (excluding the edges between PC nodes, since PCs are independent of one another) was used as the input.

edge	forward	backward	absence
rs11305802-TMEM55B	0.02	0.00	0.98
rs11305802-RP11-203M5.8	0.32	0.00	0.68
rs11305802-PNP	1.00	0.00	0.00
rs11305802-PC1	0.00	0.00	1.00
rs11305802-PC2	0.00	0.00	1.00
rs11305802-PC6	0.00	0.00	1.00
rs11305802-PC7	0.00	0.00	1.00
rs11305802-PC9	0.00	0.00	1.00
TMEM55B-RP11-203M5.8	0.34	0.51	0.16
TMEM55B-PNP	0.06	0.94	0.00
TMEM55B-PC1	0.15	0.00	0.85
TMEM55B-PC2	0.00	0.00	1.00
TMEM55B-PC6	0.00	0.00	1.00
TMEM55B-PC7	0.00	0.00	1.00
TMEM55B-PC9	0.00	0.04	0.96
RP11-203M5.8-PNP	0.22	0.78	0.00
RP11-203M5.8-PC1	0.00	0.00	1.00
RP11-203M5.8-PC2	0.00	0.00	1.00
RP11-203M5.8-PC6	0.34	0.26	0.40
RP11-203M5.8-PC7	0.00	0.00	1.00
RP11-203M5.8-PC9	0.00	0.00	1.00
PNP-PC1	0.00	0.01	0.99
PNP-PC2	0.00	0.00	1.00
PNP-PC6	0.00	0.00	1.00
PNP-PC7	0.00	0.00	1.00
PNP-PC9	0.00	0.00	1.00
