# Supplementary material for: Approximate Bayesian inference of directed acyclic graphs in biology with flexible priors on edge states
Source: PLoS Comput Biol. 2026 Mar 16;22(3):e1014039. doi: 10.1371/journal.pcbi.1014039 (PMC13046286; doi:10.1371/journal.pcbi.1014039)
Supplement: S11 Table — A fully connected graph (excluding the edges between PC nodes) was used as the input. (PDF) [file pcbi.1014039.s032.pdf]

S11 Table. Posterior probabilities from scanBMA on the GEUVADIS eQTL-gene set Q8 with associated PCs. A fully connected graph (excluding the edges between PC nodes, since PCs are independent of one another) was used as the input.

edge	forward	backward	absence
rs11305802-TMEM55B	0.00	0.00	NA
rs11305802-RP11-203M5.8	0.07	0.00	NA
rs11305802-PNP	1.00	0.00	NA
rs11305802-PC1	0.07	0.00	NA
rs11305802-PC2	0.00	0.00	NA
rs11305802-PC6	0.20	0.00	NA
rs11305802-PC7	0.00	0.00	NA
rs11305802-PC9	0.27	0.00	NA
TMEM55B-RP11-203M5.8	0.84	0.80	NA
TMEM55B-PNP	1.00	1.00	NA
TMEM55B-PC1	1.00	1.00	NA
TMEM55B-PC2	0.28	0.23	NA
TMEM55B-PC6	0.00	0.00	NA
TMEM55B-PC7	0.00	0.00	NA
TMEM55B-PC9	0.94	1.00	NA
RP11-203M5.8-PNP	1.00	1.00	NA
RP11-203M5.8-PC1	0.00	0.00	NA
RP11-203M5.8-PC2	0.50	0.77	NA
RP11-203M5.8-PC6	1.00	1.00	NA
RP11-203M5.8-PC7	0.12	0.00	NA
RP11-203M5.8-PC9	0.02	0.03	NA
PNP-PC1	0.19	0.68	NA
PNP-PC2	0.76	0.93	NA
PNP-PC6	0.04	0.00	NA
PNP-PC7	0.57	0.17	NA
PNP-PC9	0.61	0.96	NA
