# Supplementary material for: Approximate Bayesian inference of directed acyclic graphs in biology with flexible priors on edge states
Source: PLoS Comput Biol. 2026 Mar 16;22(3):e1014039. doi: 10.1371/journal.pcbi.1014039 (PMC13046286; doi:10.1371/journal.pcbi.1014039)
Supplement: S12 Table — A fully connected graph was used as the input. (PDF) [file pcbi.1014039.s033.pdf]

S12 Table. Posterior probabilities from baycn on the GEUVADIS eQTL-gene set Q8 without the associated PCs. A fully connected graph was used as the input.

edge	forward	backward	absence
rs11305802-TMEM55B	0.210	0.000	0.79
rs11305802-RP11-203M5.8	0.410	0.000	0.59
rs11305802-PNP	1.000	0.000	0.00
TMEM55B-RP11-203M5.8	0.525	0.465	0.01
TMEM55B-PNP	0.155	0.845	0.00
RP11-203M5.8-PNP	0.185	0.815	0.00
