# Supplementary material for: Approximate Bayesian inference of directed acyclic graphs in biology with flexible priors on edge states
Source: PLoS Comput Biol. 2026 Mar 16;22(3):e1014039. doi: 10.1371/journal.pcbi.1014039 (PMC13046286; doi:10.1371/journal.pcbi.1014039)
Supplement: S13 Table — A fully connected graph was used as the input. (PDF) [file pcbi.1014039.s034.pdf]

S13 Table. Posterior probabilities from baycn on the GEUVADIS eQTL-gene set Q20. A fully connected graph was used as the input.

edge	forward	backward	absence
rs142060986-RP11292F9.1	0	0	1
rs142060986-FAM27E1	1	0	0
RP11292F9.1-FAM27E1	0	1	0
