# Supplementary material for: Approximate Bayesian inference of directed acyclic graphs in biology with flexible priors on edge states
Source: PLoS Comput Biol. 2026 Mar 16;22(3):e1014039. doi: 10.1371/journal.pcbi.1014039 (PMC13046286; doi:10.1371/journal.pcbi.1014039)
Supplement: S14 Table — A fully connected graph was used as the input. The rows highlighted in yellow indicate the edges between the nodes of interest. (PDF) [file pcbi.1014039.s035.pdf]

S14 Table. Posterior probabilities from baycn for the GEUVADIS eQTL-gene set Q21 with two PCs are included in the network as confounding variables. A fully connected graph was used as the input. The rows highlighted in yellow indicate the edges between the nodes of interest.

edge	forward	backward	absence
rs147156488-FAM27C	0.000	0.000	1.000
rs147156488-FAM27A	0.000	0.000	1.000
rs147156488-FAM27D1	1.000	0.000	0.000
rs147156488-PC1	1.000	0.000	0.000
rs147156488-PC3	1.000	0.000	0.000
FAM27C-FAM27A	0.510	0.030	0.460
FAM27C-FAM27D1	0.000	1.000	0.000
FAM27C-PC1	0.250	0.030	0.720
FAM27C-PC3	0.180	0.140	0.680
FAM27A-FAM27D1	0.000	0.510	0.490
FAM27A-PC1	0.145	0.050	0.805
FAM27A-PC3	0.555	0.115	0.330
FAM27D1-PC1	0.245	0.220	0.535
FAM27D1-PC3	0.160	0.125	0.715
