# Supplementary material for: Approximate Bayesian inference of directed acyclic graphs in biology with flexible priors on edge states
Source: PLoS Comput Biol. 2026 Mar 16;22(3):e1014039. doi: 10.1371/journal.pcbi.1014039 (PMC13046286; doi:10.1371/journal.pcbi.1014039)
Supplement: S15 Table — A fully connected graph was used as input. The rows highlighted in yellow indicate the edges between the nodes of interest. (PDF) [file pcbi.1014039.s036.pdf]

S15 Table. Posterior probabilities from baycn on the GEUVADIS eQTL-gene set Q23 with two PCs included in the network as confounding variables. A fully connected graph was used as input. The rows highlighted in yellow indicate the edges between the nodes of interest.

edge	forward	backward	absence
rs150605045-AGAP9	1.000	0.000	0.000
rs150605045-AGAP10	1.000	0.000	0.000
rs150605045-PC1	1.000	0.000	0.000
rs150605045-PC8	1.000	0.000	0.000
AGAP9-AGAP10	0.720	0.280	0.000
AGAP9-PC1	0.595	0.385	0.020
AGAP9-PC8	0.495	0.455	0.050
AGAP10-PC1	0.065	0.065	0.870
AGAP10-PC8	0.035	0.080	0.885
