# Supplementary material for: Approximate Bayesian inference of directed acyclic graphs in biology with flexible priors on edge states
Source: PLoS Comput Biol. 2026 Mar 16;22(3):e1014039. doi: 10.1371/journal.pcbi.1014039 (PMC13046286; doi:10.1371/journal.pcbi.1014039)
Supplement: S16 Table — A fully connected graph was used as input. The rows highlighted in yellow indicate the edges between the nodes of interest. (PDF) [file pcbi.1014039.s037.pdf]

S16 Table. Posterior probabilities from baycn on the GEUVADIS eQTL-gene set Q37 with one PC included in the network as a confounding variable. A fully connected graph was used as input. The rows highlighted in yellow indicate the edges between the nodes of interest.

edge	forward	backward	absence
rs3858954-GOLGA6L19	0.660	0.000	0.340
rs3858954-GOLGA6L9	0.335	0.000	0.665
rs3858954-GOLGA6L20	0.965	0.000	0.035
rs3858954-PC1	0.100	0.000	0.900
GOLGA6L19-GOLGA6L9	0.035	0.055	0.910
GOLGA6L19-GOLGA6L20	0.335	0.665	0.000
GOLGA6L19-PC1	0.665	0.210	0.125
GOLGA6L9-GOLGA6L20	0.175	0.825	0.000
GOLGA6L9-PC1	0.110	0.030	0.860
GOLGA6L20-PC1	0.135	0.050	0.815
