# Supplementary material for: Approximate Bayesian inference of directed acyclic graphs in biology with flexible priors on edge states
Source: PLoS Comput Biol. 2026 Mar 16;22(3):e1014039. doi: 10.1371/journal.pcbi.1014039 (PMC13046286; doi:10.1371/journal.pcbi.1014039)
Supplement: S17 Table — A fully connected graph was used as input. The rows highlighted in yellow indicate the edges between the nodes of interest. (PDF) [file pcbi.1014039.s038.pdf]

S17 Table. Posterior probabilities from baycn on the GEUVADIS eQTL-gene set Q50 with two PCs included in the network as confounding variables. A fully connected graph was used as input. The rows highlighted in yellow indicate the edges between the nodes of interest.

| edge | forward | backward | absence |
|---|---|---|---|
| rs7124238-SBF2-AS1 | 0.195 | 0.000 | 0.805 |
| rs7124238-SWAP70 | 1.000 | 0.000 | 0.000 |
| rs7124238-PC1 | 0.135 | 0.000 | 0.865 |
| rs7124238-PC5 | 0.465 | 0.000 | 0.535 |
| SBF2-AS1-SWAP70 | 0.035 | 0.965 | 0.000 |
| SBF2-AS1-PC1 | 0.035 | 0.020 | 0.945 |
| SBF2-AS1-PC5 | 0.015 | 0.040 | 0.945 |
| SWAP70-PC1 | 0.550 | 0.415 | 0.035 |
| SWAP70-PC5 | 0.280 | 0.720 | 0.000 |
