# Supplementary material for: Approximate Bayesian inference of directed acyclic graphs in biology with flexible priors on edge states
Source: PLoS Comput Biol. 2026 Mar 16;22(3):e1014039. doi: 10.1371/journal.pcbi.1014039 (PMC13046286; doi:10.1371/journal.pcbi.1014039)
Supplement: S18 Table — A fully connected graph was used as input. (PDF) [file pcbi.1014039.s039.pdf]

S18 Table. Posterior probabilities from baycn on the GEUVADIS eQTL-gene set Q62. A fully connected graph was used as input.

edge	forward	backward	absence
rs9426902-S100A6	0.845	0.00	0.155
rs9426902-S100A4	0.420	0.00	0.580
S100A6-S100A4	0.720	0.28	0.000
